# A Purely Biomanufactured System for Delivering Nanoparticles and STING Agonists

**DOI:** 10.1002/advs.202408539

**Published:** 2024-11-26

**Authors:** Yu‐an Li, Yi Feng, Wenjing Li, Yuqin Zhang, Yanni Sun, Shifeng Wang, Roy Curtiss, Huoying Shi

**Affiliations:** ^1^ College of Veterinary Medicine Yangzhou University Yangzhou Jiangsu 225009 China; ^2^ Jiangsu Co‐innovation Center for the Prevention and Control of Important Animal Infectious Diseases and Zoonoses Yangzhou China; ^3^ Department of Infectious Diseases and Immunology College of Veterinary Medicine University of Florida Gainesville FL 32611‐0880 USA; ^4^ Joint International Research Laboratory of Agriculture and Agri‐Product Safety Yangzhou University (JIRLAAPS) Yangzhou China

**Keywords:** nanoparticle, purely biological system, STING agonist, universal vaccine

## Abstract

Nanovaccines have significantly contributed in the prevention and treatment of diseases. However, most of these technologies rely on chemical or hybrid semibiological synthesis methods, which limit the manufacturing performance advantages and improved inoculation outcomes compared with traditional vaccines. Herein, a universal and purely biological nanovaccine system is reported. This system integrates three modules: (1) self‐assembling nanoparticles, (2) self‐catalyzed synthesis of small‐molecule stimulator of interferon gene (STING) agonists, and (3) delivery vectors that target the cytosolic surveillance system. Various nanoparticles are efficiently self‐assembled using this system. After confirming the excellent immunostimulatory and lymph node targeting of this system, its broad‐spectrum antiviral efficacy is further demonstrated. By leveraging the comprehensive biosynthetic capabilities of bacterial cells, this system can efficiently combine various adjuvant‐active modular components and antigenic cargo, thereby providing a highly diversified and potent vaccine platform.

## Graphical abstract

The PBNV system provides extensive antiviral effectiveness via the following mechanisms: (1) self‐assembly of diverse nanoparticles; (2) self‐catalyzed production of small‐molecule STING agonists; and (3) delivery vectors aimed at the cytosolic surveillance system. The system enhances the periplasmic targeting of cargos, hence improving the self‐assembly of nanoparticles. Furthermore, the system biosynthesizes STING agonists and demonstrates lymph node targeting in vivo.

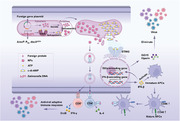

## 1 Introduction

Advancements in biomedical technology, particularly the introduction of nano‐and microscale techniques, have greatly propelled vaccine development.^[^ ^1^, ^2^ ^]^ Well‐designed multifunctional nanovaccines can improve immune responses by enhancing various dimensions of immune activation pathways, such as boosting cellular uptake,^[^ ^3^, ^4^ ^]^ targeting antigen‐presenting cells (APCs) or the lymphatic system,^[^ ^3^ ^]^ or enhancing escape from endosomes.^[^ ^5^ ^]^ However, current nanovaccine technologies rely on chemical or hybrid semibiological synthesis methods,^[^ ^6^, ^7^ ^]^ which significantly limit production efficiency. In contrast, engineered bacteria and mammalian cells can achieve complete protein biosynthesis. Several promising protein‐based nanovaccines have been developed, including virus‐like particles, 60‐mer nanoparticles (NPs), ferritin, and capsomeres.^[^ ^5^, ^8^, ^9^, ^10^ ^]^ However, most protein‐based nanovaccines require specific assembly conditions and buffer systems, which increase production costs and prolong production cycles.^[^ ^11^, ^12^ ^]^ Therefore, developing a more integrated and efficient nanovaccine production platform that meets the growing demand for vaccinations is urgently needed.

Antigen‐specific vaccines generally target only a single pathogen or species, significantly limiting their universality.^[^ ^13^ ^]^ In the context of emerging infectious diseases, extended development timelines and inherent limitations of vaccines can result in uncontrolled disease propagation and significant mortality.^[^ ^14^ ^]^ Thus, developing broad‐spectrum universal vaccines to address the continuous emergence of infectious pathogens is crucial. Recently, antiviral therapies that leverage the innate immune response have attracted considerable interest.^[^ ^15^ ^]^ The activation of innate immunity associated with pattern recognition receptors (PRRs) not only induces the expression of interferon‐stimulating genes (ISGs) that directly target the virus,^[^ ^16^ ^]^ but also enhances the maturation of APCs,^[^ ^17^ ^]^ promotes T‐cell activation,^[^ ^17^ ^]^ and induces trained immunity.^[^ ^18^ ^]^

Cytoplasmic nucleic acids stimulating the cytosolic cyclic GMP‐AMP synthase (cGAS)‐STING axis can activate interferon regulatory factor 3 (IRF3) and nuclear factor‐κB (NF‐κB), promoting the transcription of type I interferons (IFNs) and other proinflammatory cytokines to enhance antigen presentation and immune responses.^[^ ^19^ ^]^ Therefore, STING agonists have garnered significant attention in vaccine development and antiinfective therapies.^[^ ^20^ ^]^ Recent studies have shown that diadenylate cyclase (DacA) from gram‐positive bacteria can catalyze the formation of cyclic di‐AMP (CDA) from two ATP molecules.^[^ ^21^ ^]^ As the structure of CDA is similar to that of cyclic GMP‐AMP (cGAMP), it can also be recognized by STING, thereby activating downstream innate immune pathways.^[^ ^22^ ^]^ CDA is involved in the innate immune response.^[^ ^23^ ^]^ In addition, CDA has an immunomodulatory function and has been shown to improve the outcome of cancer therapy.^[^ ^24^ ^]^ However, whether CDA possesses vaccine adjuvant activity remains unclear.

STING is widely distributed in various types of host cells such as innate immune cells,^[^ ^25^ ^]^ endothelial cells,^[^ ^26^ ^]^ and T‐cells,^[^ ^27^ ^]^ providing a basis for the local administration of STING agonists. As STING is a receptor located in the cytoplasm of host cells,^[^ ^24^ ^]^ exogenous STING agonists need to traverse the host cell membrane and enter the cytoplasm to activate it.^[^ ^20^ ^]^ However, as a negatively charged hydrophilic molecule, CDA cannot autonomously penetrate or cross cell membranes.^[^ ^23^ ^]^ Despite the development of cationic adjuvants for assisting the entrance of STING agonists in cells,^[^ ^20^ ^]^ “off‐target” effects associated with cationic adjuvant‐encapsulated STING agonists may occur. For instance, STING activation in effector T‐cells can lead to cell apoptosis, ultimately hindering the development of antiviral immune responses.^[^ ^28^ ^]^ Thus, developing more efficient and targeted delivery strategies for STING agonists and CDA is crucial.

*Salmonella* spp. target the cytosolic surveillance pathway of innate immune cells.^[^ ^29^, ^30^ ^]^ Recombinant attenuated *Salmonella* vectors have been employed for the delivery of antigens, enzymes, and polysaccharides, among other cargo.^[^ ^31^ ^]^ However, the expression levels of foreign antigens delivered by *Salmonella* vectors are low, thus failing to effectively stimulate the host immune system.^[^ ^32^ ^]^ Additionally, foreign antigens or cargo produced in the cytoplasm of *Salmonella* cells are constrained by their own membrane structures, impeding their release and binding to receptors in the host cell cytoplasm, and thereby hindering the development of downstream immune responses.^[^ ^32^ ^]^ To address the aforementioned deficiencies of *Salmonella* vectors, we previously developed a *Salmonella*‐based delivery system based on MazF endoribonuclease regulation, termed the *Salmonella* mRNA interferase regulation vector (SIRV) system.^[^ ^33^ ^]^ This system mediates the enhancement of foreign antigen expression in *Salmonella* cells while actively creating membrane pores to release intracellular substances by differentially regulating mRNA degradation with or without ACA base triplets.^[^ ^33^ ^]^ In this study, we used *Salmonella* strains expressing the SIRV system as vectors for developing a new purely biomanufactured nanoparticle vector (PBNV) system. This new system improved the periplasmic targeting of cargo, thereby enhancing the self‐assembly of NPs. In addition, it simultaneously biosynthesized STING agonists, specifically targeting them for exposure to the host innate immune system. Upon in vivo delivery of NPs and STING agonists, potent immune stimulation and broad‐spectrum antiviral effects were confirmed.

## 2 Results

### 2.1 The PBNV System Enhanced the Synthesis Efficiency and Periplasmic Targeting Secretion Efficiency of The Desired Protein

**Scheme 1:**
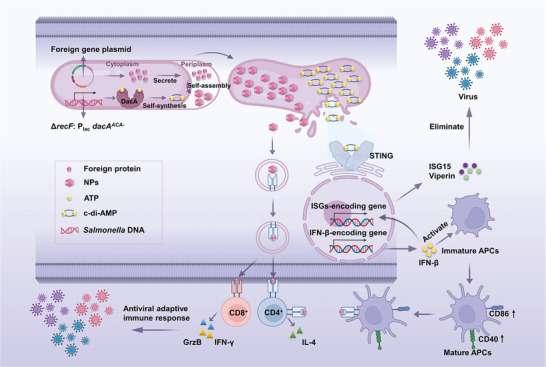
Diagram of the model illustrating the PBNV system. The platform consists of two modules. (1) Self‐assembling nanoparticle antigens: A plasmid containing a foreign gene is introduced into the *Salmonella* vector. Under the control of the SIRV system, this gene is highly expressed and secreted into the periplasm, where it efficiently self‐assembles into NPs. (2) Biosynthesis of the STING agonist c‐di‐AMP (CDA): The *dacA* gene is integrated into the recombinant *Salmonella* genome, encoding a diadenylate cyclase that catalyzes the conversion of ATP into the STING agonist CDA, providing adjuvant activity. The substantial release of CDA from the SIRV vector activates the STING‐IFN‐β‐ISGs axis of the host cell, inducing a broad‐spectrum antiviral response. The activation of the STING pathway enhances the activation of APCs and T‐cells, thereby improving antiviral adaptive immune responses.

**Figure 1:**
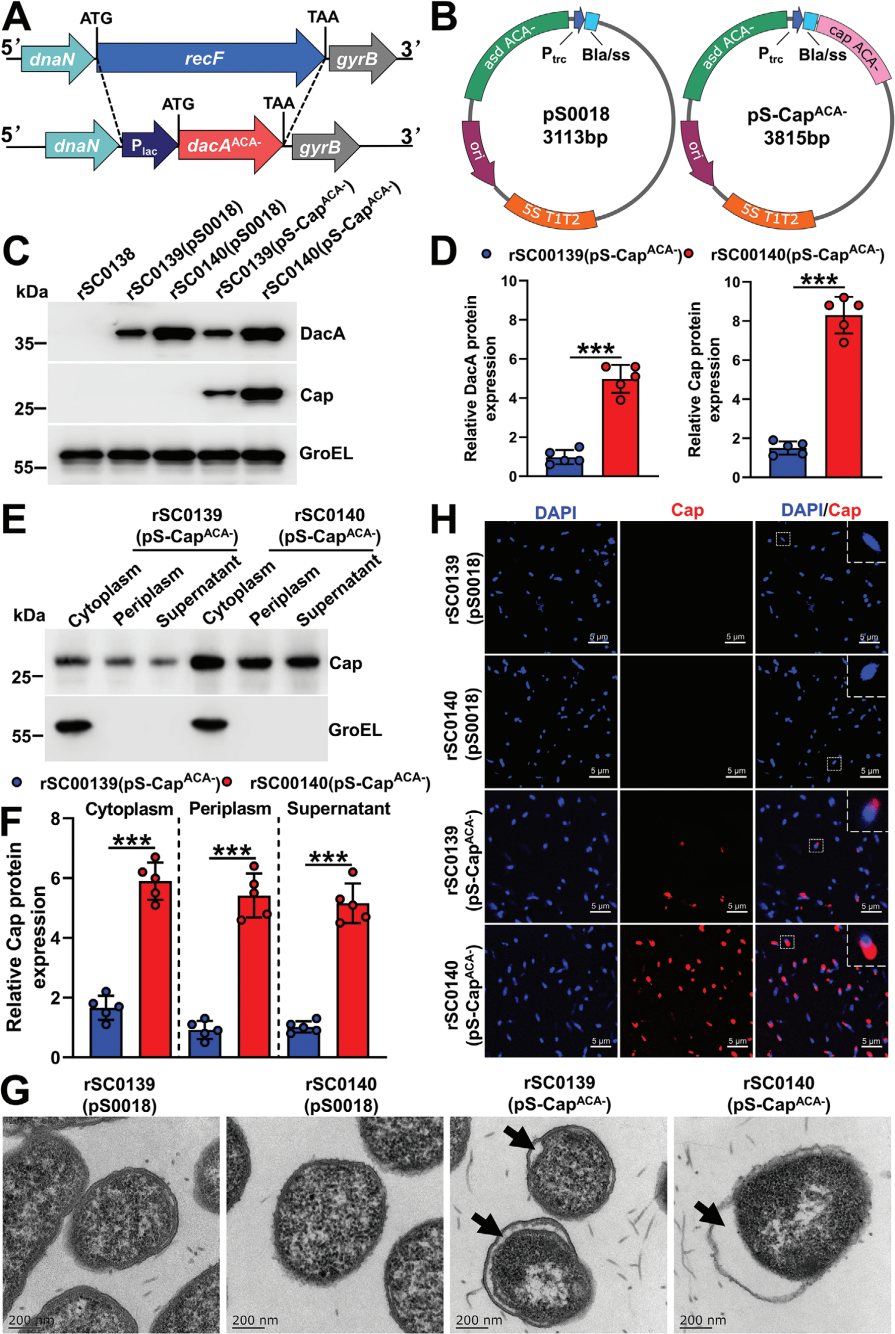
The PBNV system enhances the synthesis efficiency and periplasmic targeting secretion efficiency of the desired protein. A) Schematic map of the Δ*recF*:P_lac_ *dacA^ACA−^* deletion‐insertion mutation. B) Plasmid maps of empty vector pS0018 (left) and recombinant plasmid pS‐Cap^ACA‐^ (right). The synthesis of DacA and Cap in *Salmonella* strains was analyzed using western blotting with DacA‐ and Cap‐specific antibodies and normalized to the expression of GroEL; A representative image C) and densitometric measurements D) of DacA and Cap are shown. Subcellular localization analyses of Cap expressed in *Salmonella*. A representative image E) and densitometric measurements F) are shown. The synthesis of Cap in the cytoplasm, periplasm, and supernatant was analyzed using western blotting with a Cap‐specific antibody and normalized to the expression of GroEL. G) TEM images of control and nanoparticle‐expressing cells (bars = 200 nm). H) The expressed proteins were also detected by immunofluorescence (bars = 5 µm). (C‐F) n = five biological replicates per group; one representative picture is shown for each group. (D, F) ***, *P* < 0.001. The experiments were performed twice. The results from both experiments were similar, and the data were pooled for analysis.

We envisioned the employment of bacterial vectors for the self‐assembly of protein‐based NPs and biosynthesis of STING agonists, incorporating the concept of in vivo self‐synthesis of nanovaccines and innate immune stimulants using a recently reported SIRV strategy.^[^ ^33^ ^]^ Specifically, foreign NPs and *dacA* genes were introduced into the *Salmonella* vector either in the form of a plasmid or through chromosomal mutations. Under the actions of PBNV and secretion signal peptides, abundant foreign proteins were produced in the bacterial cytoplasm and secreted into the periplasmic space, where they efficiently folded and assembled into NPs. DacA was also efficiently synthesized under the action of the PBNV system, converting ATP present in bacterial cells into CDA (**Scheme** **1**). *recF* was replaced with a P_lac_ *dacA* ^ACA−^ cassette (**Figure** **1**A). The porcine circovirus type 2 *capsid* (PCV2‐*cap*),^[^ ^34^ ^]^ human papillomavirus type 16 capsid (HPV‐*l1*),^[^ ^35^ ^]^ and B subunits of the cholera toxin C‐terminal trimer‐forming peptide (*ctbtri*)^[^ ^12^ ^]^ genes without ACA base triplets were cloned into plasmid pS0018 to generate the pS‐Cap^ACA−^, pS‐L1^ACA−^, and pS‐CTBTtri^ACA−^ plasmids (Figure 1B; Figure S1A, Supporting Information). The expression levels of DacA, Cap, L1, and CTBTri in strain rSC0140 carrying the PBNV system were significantly higher than those in strain rSC0139 (no PBNV system) (Figure 1C,D; Figure S1B,C, Supporting Information), indicating that the PBNV system can significantly upregulate the expression of DacA and Cap. The periplasmic space of bacteria facilitates the soluble expression and correct folding of proteins owing to the unique oxidative environment,^[^ ^36^ ^]^ thereby promoting nanoparticle assembly.^[^ ^12^ ^]^ In addition to Cap, we selected two proteins derived from viruses or bacteria to analyze their subcellular localization in PBNV.^[^ ^12^, ^35^ ^]^ Due to the effect of the periplasmic‐targeting signal peptide Bla/SS,^[^ ^37^ ^]^ Cap, L1, and CTBTri exhibited periplasmic and supernatant secretion in both the rSC0140 and rSC0139 strains (Figure 1E,F; Figure S1, Supporting Information). Notably, the expression levels of Cap, L1, and CTBTri in the periplasm and supernatant of rSC0140 were significantly higher than those in rSC0139 (Figure 1E,F; Figure S1B,C, Supporting Information). These results suggested that the PBNV system improves the periplasmic targeting of foreign proteins. Transmission electron microscopy (TEM) analysis revealed significant morphological differences in cells expressing the Cap protein compared with the same strain without Cap expression (Figure 1G). Specifically, cells expressing Bla/SS‐Cap fusion monomers exhibited a pronounced expansion in the gap between their inner and outer membranes, clearly indicating the successful expression and localization of the designed NPs within the bacterial periplasmic space (Figure 1G). Furthermore, the gap between the inner and outer membranes of rSC0140(pS‐Cap^ACA−^), which carries the PBNV system, was significantly larger than that of rSC0139(pS‐Cap^ACA−^), which does not contain the PBNV system (Figure 1G), suggesting that the PBNV system and Bla/SS signal peptide synergistically promote the periplasmic targeting of NPs. The presence and localization of fusion monomers within cells were confirmed using Cap‐specific monoclonal antibodies and laser scanning confocal microscopy (LSCM) (Figure 1H). Furthermore, the intensity of the Cap‐specific signal (red) in rSC0140(pS‐Cap^ACA−^) was significantly higher than that in rSC0139(pS‐Cap^ACA−^), indicating that the PBNV system enhanced the periplasmic targeting of foreign proteins (Figure 1H).

### 2.2 The PBNV System Enhanced the Yield, Release, and In Vivo Delivery of NPs

**Figure 2:**
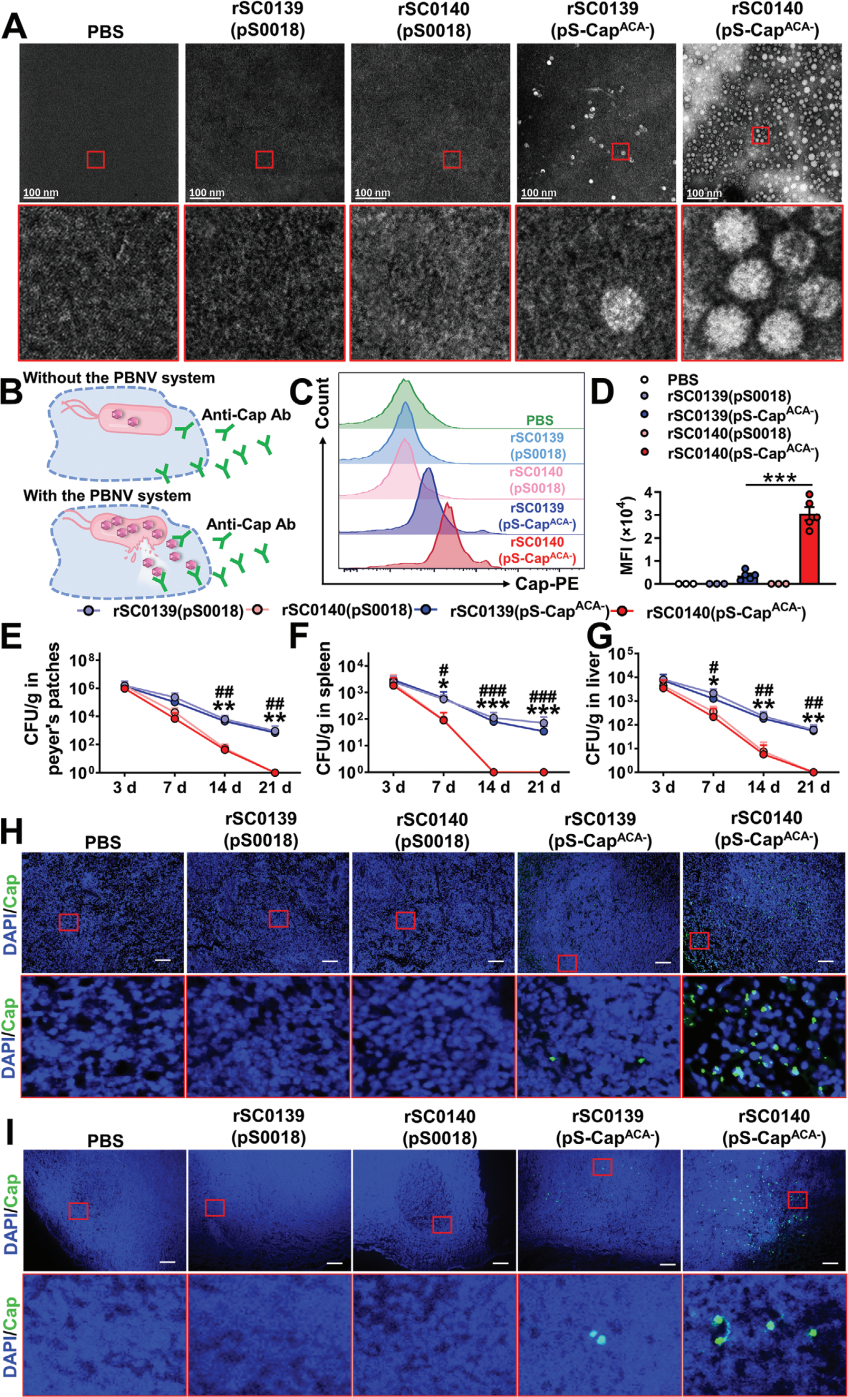
The PBNV system enhances the synthesis and host delivery efficiency of Cap‐NPs. A) TEM analyses of PCV2‐VLPs from lysates of PBS, rSC0139(pS0018), rSC0140(pS0018), rSC0139(pS‐Cap^ACA−^), or rSC0140(pS‐Cap^ACA−^) (bars = 100 nm). The red box indicates Cap‐NPs. B) Selective permeabilization of BMDCs membranes enabled the detection of released Cap at 12 h after infection of BMDC with strains rSC0139 and rSC0140, either with pS0018 or pS‐Cap^ACA−^. The mean fluorescence intensity (MFI) of Cap in the cytoplasm of BMDCs was quantified using flow cytometry. Representative images C) and statistical analysis histograms D) are shown. Colonization in Peyer's patch E), spleen F), and liver G) of mice on days 3, 7, 14, and 21 with the indicated strains after oral inoculation with 10^9^ CFU bacteria. Of note, 10 CFU per Peyer's patch or gram of tissue was set as the limit of detection of the assay. H) Immunofluorescence analysis in mice. Mice were orally inoculated with rSC0139(pS0018), rSC0140(pS0018), rSC0139(pS‐Cap^ACA−^), rSC0140(pS‐Cap^ACA−^), and PBS. Spleen were isolated from mice 3 d after inoculation. Paraffin sections of tissues were stained for Cap (green). DAPI (blue) indicates the nuclei, and red boxes highlight particles that bind to Cap‐specific antibodies (bars = 100 µm). I) Immunofluorescence analysis in pigs. Pigs were orally inoculated with rSC0139(pS0018), rSC0140(pS0018), rSC0139(pS‐Cap^ACA−^), rSC0140(pS‐Cap^ACA−^), and PBS. Inguinal lymph nodes were isolated from pigs 3 d after inoculation. Paraffin sections of inguinal lymph nodes were stained for Cap (green). DAPI (blue) indicates the nuclei, and red boxes highlight particles that bind to Cap‐specific antibodies (bars = 100 µm). (A, C, D, H, I) n = five biological replicates per group. One representative picture is shown for each staining and treatment group. (E‐G) n = 10 mice. Data are expressed as the mean ± SEM. *P* values were calculated using one‐way ANOVA with Tukey's multiple comparison test. Asterisks indicate significant differences between groups linked by horizontal lines. (D) ***, *P* < 0.001. (E‐G) ###, *P* < 0.001; ##, *P* < 0.01; #, *P* < 0.05, compared with strains rSC0139(pS0018) and rSC0140(pS0018); ***, *P* < 0.001; **, *P* < 0.01; *, *P* < 0.05, compared with strains rSC0139(pS‐Cap^ACA−^) and rSC0140(pS‐Cap^ACA−^). The experiments were performed twice. The results from both experiments were similar, and the data were pooled for analysis.

The Cap‐NPs are assembled from 60 copies of Cap proteins with the icosahedral symmetry and several exposed loops located on the viral surface, which may serve as the immunodominant epitopes eliciting neutralization antibodies.^[^ ^34^ ^]^ The L1‐NPs are arranged in a *T* = 7 icosahedral lattice, comprises of 72 pentameric capsomeres, assembled from 360 copies of L1.^[^ ^35^ ^]^ The monomer of the CTBTri‐NP contains an 103‐aa B_5_ subunits of cholera toxin (CTB), a C‐terminal trimer‐forming peptide, and a 29‐aa glycosylation sequence.^[^ ^12^ ^]^ The addition of the C‐terminal trimer‐forming peptide causes the bioconjugate to conglomerate into a quaternary spherical structure with protein at the core.^[^ ^12^ ^]^ Once we verified that modular fusion monomers were activated to produce proteins within the cell and gathered specifically in the periplasmic region, we ruptured *Salmonella* cells and examined the properties of NPs in the resulting lysate using transmission electron microscopy (TEM) and dynamic light scattering (DLS). The lysates obtained from the rSC0139(pS0018) and rSC0140(pS0018) strains, which did not express any foreign proteins, did not contain any visible particles. Whereas, the rSC0139(pS‐Cap^ACA−^) and rSC0140(pS‐Cap^ACA−^) foreign protein‐delivery strains produced NPs with a diameter of ≈17 nm (**Figure** **2**A; Figure S2D, Supporting Information). Similarly, in strains delivering L1 or CTBTri, NPs had diameters of ≈53 and 25 nm, respectively (Figure S2A,B,D, Supporting Information). Notably, the yield of these NPs in the rSC0140 strain was significantly higher than that in the rSC0139 strain (Figure S2C, Supporting Information). These results indicated that the PBNV system can increase the yield of NPs in bacterial vectors, regardless of whether they originate from viruses or bacteria and irrespective of their differing diameters. We then examined the presence of foreign NPs in the cytoplasm of host cells infected with the corresponding strains (Figure 2B). We observed a significantly higher Cap‐NP signal intensity in the cytoplasm of host cells treated with rSC0140(pS‐Cap^ACA−^) compared with that in cells treated with rSC0139(pS‐Cap^ACA−^) (Figure 2C,D), indicating that PBNV enhanced the release of foreign NPs in host cells. The programmed lysis characteristics of the SIRV system promoted bacterial clearance in vivo, providing enhanced safety.^[^ ^33^ ^]^ We observed that the colonization levels of the PBNV‐carrying strain rSC0140(pS‐Cap^ACA−^) in the Peyer's patch, spleen, and liver of mice were significantly lower than those of the non‐PBNV‐carrying strain rSC0139(pS‐Cap^ACA−^). Strain rSC0140 was cleared earlier than strain rSC0139, indicating that the PBNV system bestowed the safety of the SIRV system (Figure 2E‐G). To further evaluate the efficiency of PBNV in delivering NPs in vivo, particularly to the lymphatic system, we measured the Cap‐NP signal in the spleens of mice (Figure 2H) and inguinal lymph nodes of pigs (Figure 2I). We found that the Cap‐NP signal intensity in the spleens of mice and inguinal lymph nodes of piglets inoculated with rSC0140(pS‐Cap^ACA−^) was significantly higher than that in those treated with rSC0139(pS‐Cap^ACA−^) (Figure 2H,I). These data indicated that the PBNV system significantly improved the in vivo delivery of NPs.

### 2.3 The PBNV Self‐Catalyzed the Synthesis and Release of CDA, Inducing A Broad‐Spectrum Antiviral Response

**Figure 3:**
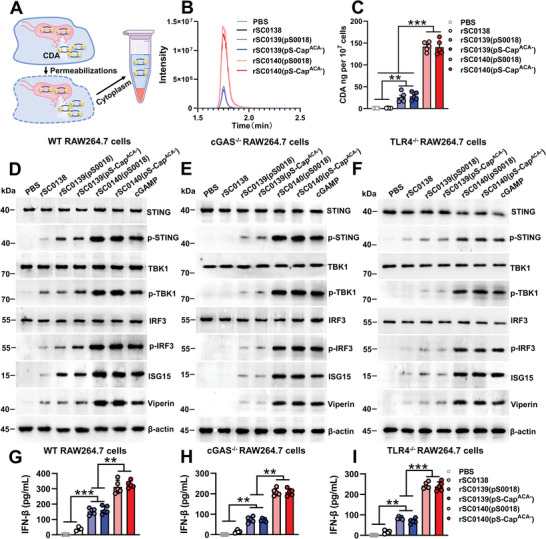
The PBNV targets CDA to the host cytoplasm, inducing activation of the STING‐ISGs axis and an antiviral response. A) Selective permeabilization of RAW 264. 7 cell membranes enabled the detection of released CDA. At the indicated time after infection with strains rSC0139 and rSC0140 carrying either pS0018 or pS‐Cap^ACA−^, CDA in RAW 264.7 cytosols were quantified using LC‐MS. The representative phenograms B) and histographs C) are shown. STING, p‐STING, TBK1, p‐TBK1, IRF3, p‐IRF3, ISG15, and viperin expression in WT RAW264.7 D), cGAS KO RAW264.7 E), and TLR4 KO RAW264.7 F) cells were assessed using western blotting at 12 h after infection with the indicated strains. For all western blot analyses, β‐actin was used as a loading control. Secretory IFN‐β levels were assessed 12 h after infection in WT RAW264.7 G), cGAS KO RAW264.7 H), and TLR4 KO RAW264.7 I) cells using ELISA. (B‐I) n = five biological replicates per group. (D‐F) n = three biological replicates per group; one representative picture is shown for each group. Data are expressed as the mean ± SEM. *P* values were calculated using one‐way ANOVA with Tukey's multiple comparison test. Asterisks indicate significant differences between groups linked by horizontal lines. (C, G‐I) ***, *P* < 0.001; **, *P* < 0.01. The experiments were performed twice. The results from both experiments were similar, and the data were pooled for analysis.

To evaluate and compare the ability of the relevant bacterial strains to target and deliver CDA to the cytoplasm of host cells, we measured the CDA levels in the cytoplasm of cells treated with the respective strains at the same infection dose (**Figure** **3**A). We noticed that the levels of CDA in the cytoplasm of cells treated with the PBNV‐carrying strain rSC0140(pS‐Cap^ACA−^) were significantly higher than those in cells treated with the non‐PBNV‐expressing rSC0139(pS‐Cap^ACA−^) (Figure 3B,C), indicating that the PBNV system enhanced the cytoplasmic delivery of CDA. We further explored the STING‐ISG axis in mouse macrophages. Apart from wild‐type RAW264.7 cells, we employed *Cgas* and toll‐like receptor 4 (*Tlr4*) knockout cells as controls. This approach allowed us to exclude interference with the IFN‐ISG pathway, which could result from the innate activation of the cGAS and TLR4 pathways by *Salmonella*.^[^ ^29^, ^30^ ^]^ The expression levels of p‐STING induced by rSC0139 and rSC0140, which produce CDA, were significantly higher than those induced by rSC0138, which does not produce CDA (Figure 3D‐F). These data indicated that *Salmonella* vectors that synthesize and deliver foreign CDA can significantly induce STING activation. Further analysis revealed that in wild‐type cells treated with rSC0140, the expression levels of p‐STING, p‐TBK1, and p‐IRF3 were significantly higher compared with those in cells treated with rSC0139 (Figure 3D). Similar phenotypes were observed in *Cgas* and *Tlr4* knockout cells, where the activation of p‐STING, p‐TBK1, and p‐IRF3 was unaffected by the lack of cGAS or TLR4 (Figure 3E,F). These results indicated that PBNV‐expressing strains significantly enhanced the activation of the downstream TBK1‐IRF3 pathway in a STING‐dependent manner. The levels of IFN‐β, ISG15, and viperin induced by rSC0139 and rSC0140 were significantly higher than those induced by rSC0138 (Figure 3D‐I). Additionally, in wild‐type cells treated with rSC0140, the expression levels of IFN‐β, ISG15, and viperin were significantly higher compared with that in cells treated with rSC0139 (Figure 3D‐I). These data indicated that synthesizing and delivering foreign CDA imparts *Salmonella* vectors with the phenotype of inducing antiviral effector responses and that the PBNV system enhances this phenotype. In wild‐type cells treated with rSC0139 and rSC0140, the titers of H1N1, H5N1, and H7N9 influenza A viruses were significantly lower than those in cells treated with rSC0138 (Figure S3A‐J, Supporting Information). Similar phenotypes were observed in *Cgas* or *Tlr4* knockout cells (Figure S3A‐J, Supporting Information). Additionally, in cells treated with PBNV‐expressing rSC0140, the titers of H1N1, H5N1, and H7N9 influenza A viruses were significantly lower than those in cells treated with non‐PBNV‐expressing rSC0139 (Figure S3A‐J, Supporting Information). These results indicated that synthesizing and delivering foreign CDA imparts *Salmonella* vectors with the ability to induce a broad‐spectrum antiviral response against influenza A viruses in host cells, thereby inhibiting viral replication. Furthermore, the PBNV system enhanced this response. To further explore their applicability across different hosts, we assessed the antiviral potential of the respective strains in a porcine cell model, 3D4/21 cells. Similar to the results observed in mouse cell models, the expression levels of p‐STING, p‐TBK1, p‐IRF3, viperin, ISG15, and IFN‐β induced by the CDA‐producing rSC0139 and rSC0140 strains were significantly higher compared with those in the non‐CDA‐producing rSC0138 (Figure S4A‐F, Supporting Information). Correspondingly, the viral titers of PCV2 in cells treated with rSC0139 or rSC0140 were significantly lower than those in cells treated with rSC0138 (Figure S4G‐I, Supporting Information). These data indicated that CDA‐producing *Salmonella* vectors can induce antiviral effector responses in porcine cells. Notably, PBNV‐expressing rSC0140 induced significantly higher expression levels of p‐STING, p‐TBK1, p‐IRF3, viperin, ISG15, and IFN‐β in porcine cells compared with those in non‐PBNV‐expressing rSC0139 (Figure S4A‐F, Supporting Information). Correspondingly, cells treated with rSC0140 exhibited significantly lower PCV2 viral titers than those treated with rSC0139 (Figure S4G‐I, Supporting Information). These data demonstrated that the PBNV system enhanced antiviral effector responses induced by CDA‐producing *Salmonella* vectors in porcine cells.

### 2.4 PBNV Efficiently Delivered CDA to the Host Lymphatic System, Inducing the Activation of the STING‐ISGs Pathway In Vivo

**Figure 4:**
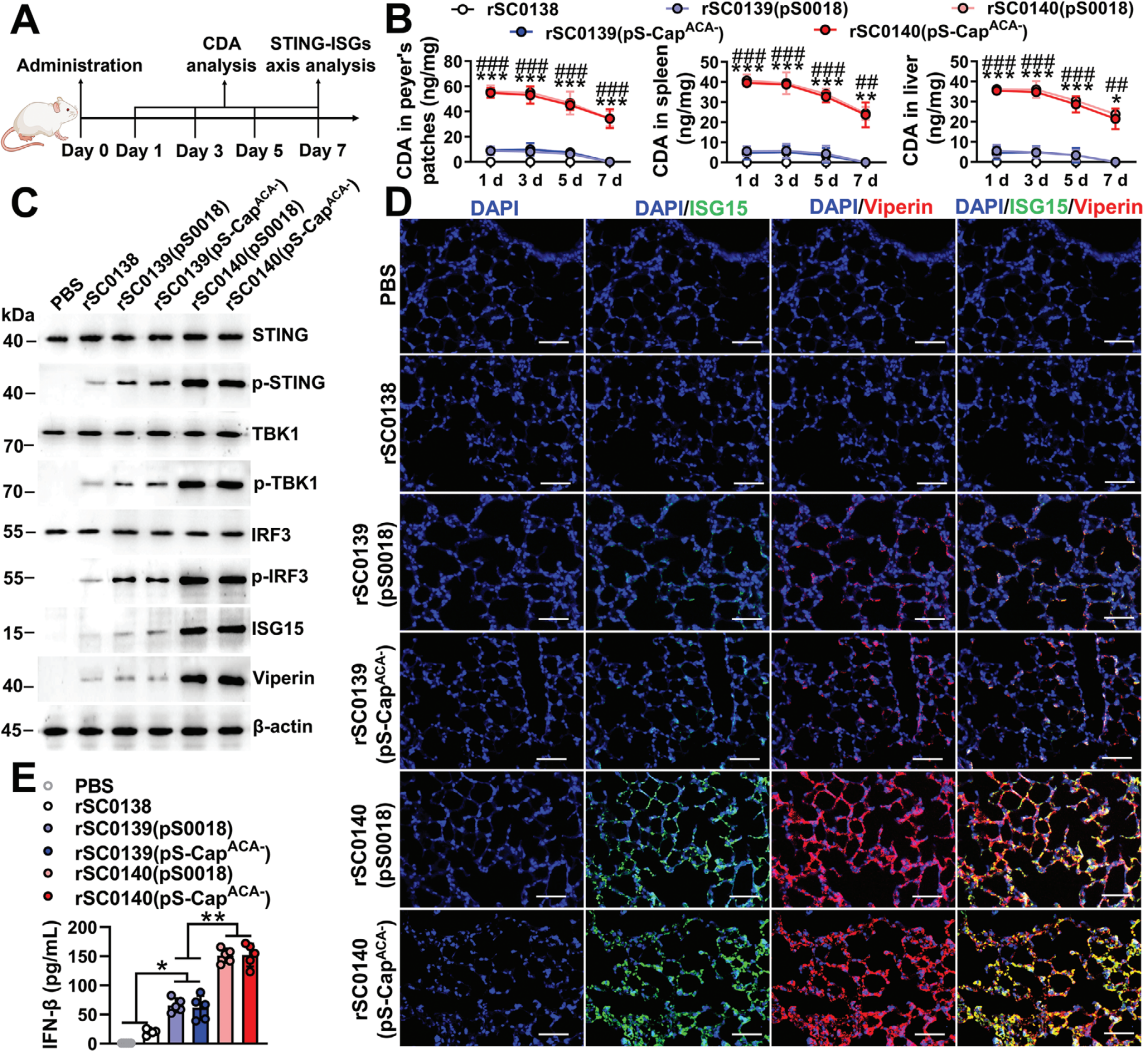
The PBNV targets CDA to the lymphatic system, inducing a systemic antiviral response. A) Administration regimen. Mice were inoculated with the relevant strains, and in vivo delivery of CDA and activation of the STING‐ISG axis were analyzed at the indicated dates after inoculation. B) CDA in the indicated tissue extracts was separated by chromatography on a C18 column and quantified using LC‐MS. C) The expression levels of STING, p‐STING, TBK1, p‐TBK1, IRF3, p‐IRF3, ISG15, and viperin in the Peyer's patches of mice were detected using western blot analysis 24 h after oral administration of the corresponding strains. D) Immunofluorescence. Mice were inoculated with the relevant strains. Lungs were isolated from mice 24 h after inoculation. Paraffin sections of the lungs were stained for viperin (red) and ISG15 (green). DAPI (blue) indicates the nuclei (bars = 50 µm). E) Secretory IFN‐β levels in mice sera were assessed using ELISA 24 h after primary immunization. (B, C, and E) n = five mice. (C) Representative images of each group. Data are expressed as the mean ± SEM. *P* values were calculated using one‐way ANOVA with Tukey's multiple comparison test. Asterisks indicate significant differences between groups linked by horizontal lines. (B) ###, *P* < 0.001; ##, *P* < 0.01, compared with strains rSC0139(pS0018) and rSC0140(pS0018); ***, *P* < 0.001; **, *P* < 0.01; *, *P* < 0.05, compared with strains rSC0139(pS‐Cap^ACA−^) and rSC0140(pS‐Cap^ACA−^). (E) **, *P* < 0.01; *, *P* < 0.05. The experiments were performed twice. The results from both experiments were similar and the data were pooled for analysis.

We detected and compared the CDA levels in tissues from mice inoculated with the strains expressing or not PBNV systems at the same dose using mass spectrometry (**Figure** **4**A). During the initial 5‐d observation period, we detected CDA in the Peyer's patches, spleens, and livers of mice inoculated with rSC0139 (Figure 4B). However, by day 7, we did not detect any CDA in these mice (< 0.5 ng mg^−1^) (Figure 4B). In contrast, we found CDA in the tissues of mice inoculated with rSC0140 throughout the 7‐d observation period (Figure 4B). The levels of CDA in Peyer's patches, spleen, and liver of mice inoculated with PBNV‐expressing rSC0140 were significantly higher than those in mice inoculated with non‐PBNV‐expressing rSC0139 (Figure 4B). These results indicated that *Salmonella* delivered exogenous CDA in vivo, and that the PBNV system significantly enhanced this efficacy. In mice and pigs inoculated with CDA‐producing rSC0139 and rSC0140, the expression levels of p‐STING, p‐TBK1, p‐IRF3, viperin, and ISG15 in lymph nodes, and IFN‐β in serum were significantly higher compared with those in animals inoculated with non‐CDA‐producing rSC0138, (Figure 4C,E; Figure S5A,C, Supporting Information). Furthermore, indirect immunofluorescence analysis revealed that in the lungs of mice and pigs inoculated with rSC0139 and rSC0140, the signal intensities of viperin (red signal) and ISG15 (green signal) were significantly higher than those in animals inoculated with rSC0138 (Figure 4D; Figure S5B, Supporting Information). These results indicated that synthesizing and delivering foreign CDA imparts the *Salmonella* vector with a significant ability to systemically induce the activation of the STING‐ISG pathway in lymphoid organs. Notably, the expression levels of IFN‐β in the serum and p‐STING, p‐TBK1, p‐IRF3, viperin, and ISG15 in the lymph nodes of mice and piglets inoculated with PBNV‐expressing rSC0140 were significantly higher than those in animals inoculated with non‐PBNV‐expressing rSC0139 (Figure 4C,E; Figure S5A,C, Supporting Information). Similarly, the signal intensities of viperin and ISG15 in the lungs of mice and pigs inoculated with rSC0140 were significantly higher than those in animals inoculated with rSC0139 (Figure 4D; Figure S5B, Supporting Information). These data indicated that the PBNV system significantly enhanced the systemic activation of the STING‐ISG pathway induced by CDA‐producing *Salmonella* vectors in vivo.

### 2.5 PBNV Induced Broad‐Spectrum Antiviral Effector Responses In Vivo

**Figure 5:**
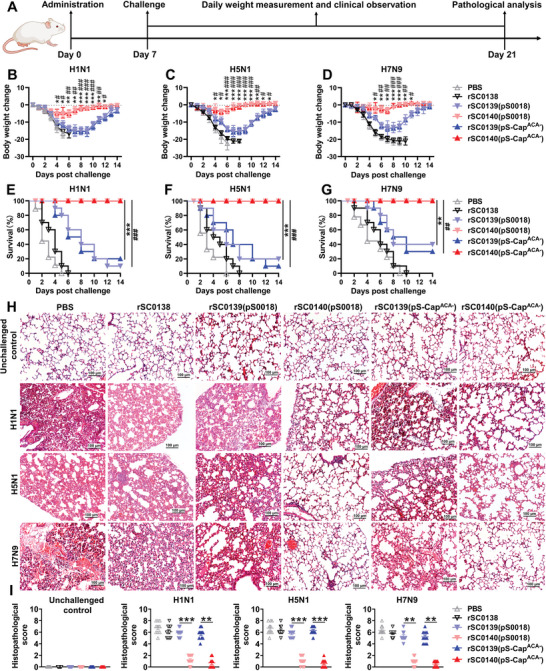
The PBNV induces a broad‐spectrum antiviral response in mice. A) Administration regimen. Body weight changes in mice challenged with H1N1 B), H5N1 C), and H7N9 D) viruses. Body weight is plotted as a percentage of the average initial weight on day 0. Body weight changes were evaluated for 14 d. Overall survival of mice challenged with H1N1 E), H5N1 F), and H7N9 G) viruses. H) Microscopic pathological observations of mice lungs after challenge with H1N1, H5N1, or H7N9. Bars = 100 µm. I) Lung histological scores (B‐G) n = 10 mice. ###, *P* < 0.001; ##, *P* < 0.01; #, *P* < 0.05, compared with strains rSC0139(pS0018) and rSC0140(pS0018); ***, *P* < 0.001; **, *P* < 0.01; *, *P* < 0.05, compared with strains rSC0139(pS‐Cap^ACA−^) and rSC0140(pS‐Cap^ACA−^). (I) ***, *P* < 0.001; **, *P* < 0.01. (B‐D, I) Data are expressed as the mean ± SEM. *P* values were calculated using one‐way ANOVA with Tukey's multiple comparison test. Asterisks indicate significant differences between groups linked by horizontal lines. (E‐G) Log‐rank test with the Holm‐Bonferroni correction. (H) n = 10 mice. A representative image of each group is shown. The experiments were performed twice. The results from both experiments were similar and the data were pooled for analysis.

To investigate whether CDA‐producing strains can play a role in controlling infection in vivo, we challenged mice nasally with influenza A virus (IAV) after treatment with the relevant strains and evaluated their protective efficacy (**Figure** **5**A). All mice in the phosphate‐buffered saline (PBS) group died within 10 d of challenge with H1N1, H5N1, or H7N9 viruses (Figure 5B). Mice administered with PBNV‐expressing rSC0140 showed significantly higher survival rates and less weight loss than mice administered with non‐PBNV‐expressing rSC0139 (Figure 5B‐G). Notably, all mice orally administered rSC0140 survived challenges with H1N1, H5N1, and H7N9, only experiencing a slight weight loss (<10%) (Figure 5B‐G). Pathological observations were consistent with the above results; the lungs of mice administered rSC0140 showed only slight microscopic lesions or no lesions, whereas those administered rSC0139 or PBS showed enhanced hemorrhage and inflammatory cell infiltration (Figure 5H,I). Correspondingly, the lung pathology scores of mice inoculated with rSC0140 were significantly lower than those of mice inoculated with rSC0139 (Figure 5H,I). Taken together, these data indicated that the PBNV‐expressing rSC0140 strain can induce broad and effective protection against IAVs in vivo and protect target tissues from viral invasion.

### 2.6 PBNV System Enhanced Antigen Presentation and T‐Cell Activation

**Figure 6:**
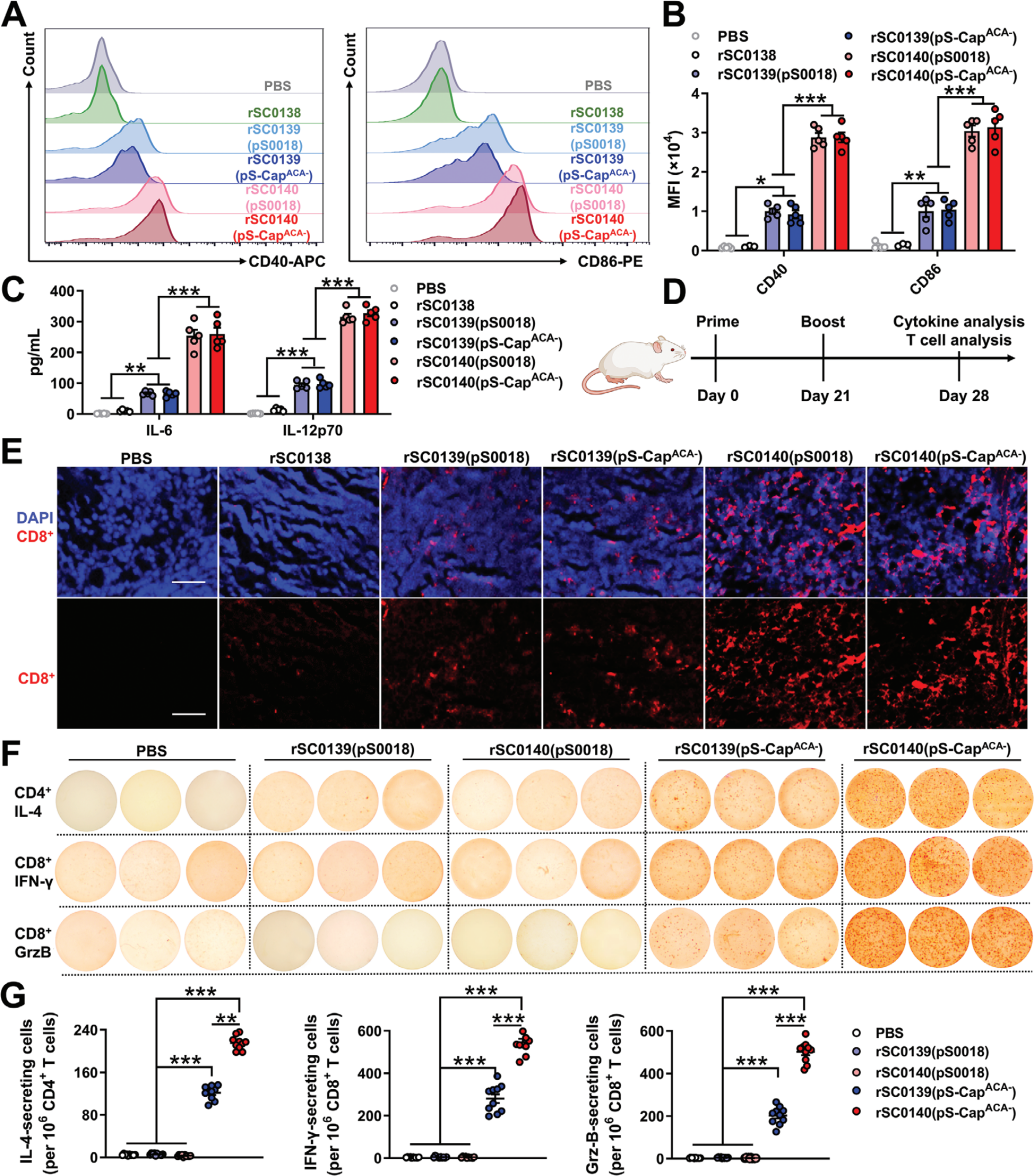
The PBNV improves APCs maturation and T cell activation. BMDCs were treated with PBS, rSC0138, rSC0139(pS0018), rSC0140(pS0018), rSC0139(pS‐Cap^ACA−^) and rSC0140(pS‐Cap^ACA−^). The MFI of CD40 and CD86 in these BMDCs were quantified using flow cytometry. Representative images A) and statistical analysis histograms B) are shown. C) Levels of IL‐6 and IL‐12p70 cytokines in BMDC supernatants; only cytokines with detectable levels are shown. D) Administration regimen. E) Immunofluorescence analysis of infiltrating CD8^+^ T‐cells in the spleen. Mice were inoculated with the relevant strains. Spleens were isolated from mice at 7 d after booster immunization. Paraffin sections of the spleen were stained for CD8^+^ (red). DAPI (blue) indicates the nuclei (bars = 50 µm). ELISPOT assay of Cap‐specific secreted cytokines from mice immunized with the relevant strains. Representative ELISPOT plate F) showing IL‐4‐producing colonies of CD4^+^ T‐cells, and IFN‐γ‐ and GrzB‐producing colonies of CD8^+^ T‐cells stimulated with Cap; the mean number of spot forming units was determined G). (B and C) n = five biological replicates per group. (E and F) n = 10 mice. Representative images from each group are shown. G) n = 10 mice. (B, C, G) Data are expressed as the mean ± SEM. *P* values were calculated using one‐way ANOVA with Tukey's multiple comparison test. Asterisks indicate significant differences between groups linked by horizontal lines. ***, *P* < 0.001; **, *P* < 0.01. The experiments were performed twice. The results from both experiments were similar and the data were pooled for analysis.

We further investigated the immunomodulatory capabilities of PBNV‐expressing strains in bone marrow‐derived dendritic cells (BMDCs) in vivo. Inoculation with CDA‐producing rSC0139 induced significantly higher expression levels of the costimulatory molecules CD40 and CD86 and the proinflammatory cytokines IL‐6 and IL‐12p70 compared with those after inoculation with non‐CDA‐producing rSC0138 (**Figure** **6**A‐C). These data indicated that the synthesis and delivery of foreign CDA imparts the *Salmonella* vector with the ability to induce APC maturation. Notably, PBNV‐expressing rSC0140 induced a significantly higher expression of CD40, CD86, IL‐6, and IL‐12p70 in BMDCs than non‐PBNV‐expressing rSC0139 (Figure 6A‐C). These findings demonstrated that the PBNV system enhanced the ability of the CDA‐producing vector to induce APC maturation. The specific fluorescence intensity of CD8^+^ T‐cells (red) was significantly higher in the spleen of mice inoculated with CDA‐producing rSC0139 than that in mice inoculated with non‐CDA‐producing rSC0138 (Figure 6E). Furthermore, the specific fluorescence intensity of CD8^+^ T‐cells in the spleen of mice inoculated with PBNV‐expressing rSC0140 was significantly higher than that in mice inoculated with non‐PBNV‐expressing rSC0139 (Figure 6E). These results indicated that synthesizing and delivering foreign CDA imparts the *Salmonella* vector with the ability to activate T‐cells, which is enhanced by the PBNV system. Next, we isolated CD4^+^ and CD8^+^ T‐cells from immunized mice and stimulated them with Cap protein to examine antigenspecific adaptive immune responses. Upon stimulation with equal doses of Cap protein, mice inoculated with rSC0140(pS‐Cap^ACA−^) showed significantly higher secretion levels of IL‐4 from CD4^+^ T‐cellsas well as IFN‐γ and granzyme B (GrzB) from CD8^+^ T‐cells than mice inoculated with rSC0139(pS‐Cap^ACA−^) (Figure 6F,G). These results indicated that the PBNV system enhanced antigen‐specific T‐cell responses.

### 2.7 PBNV System Enhanced Cellular and Humoral Immune Responses in Pigs, Preventing Viral Infections

**Figure 7:**
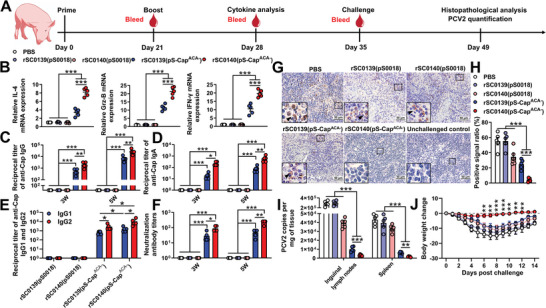
The PBNV enhances antiviral efficacy by boosting the adaptive immune response. A) Inoculation regimen. B) Cap‐specific cytokine levels in pigs immunized with the relevant strains, as determined using qRT‐PCR. Pig peripheral blood mononuclear cells (PBMCs) were activated by Cap, and the relative mRNA expression levels of IL‐4, GrzB, and IFN‐γ were determined. Serum IgG C), nasal IgA D), IgG1, and IgG2 E) responses specific to Cap, and neutralizing antibody (NAb) responses F) against PCV2 were assessed using ELISA. The data represent reciprocal antibody titers in the sera or nasal rinses from pigs orally inoculated with rSC0139(pS0018), rSC0140(pS0018), rSC0139(pS‐Cap^ACA−^), rSC0140(pS‐Cap^ACA−^), or PBS at three or five weeks after primary immunization. G) Differences in the signal intensity of PCV2 antigen in the inguinal lymph nodes detected using immunohistochemical (IHC) staining 14 d after PCV2 challenge (bars = 50 µm); PCV2‐specific signals are shown with a black box. H) The ratio of brown signals (PCV2 signals) in 100 cells was randomly calculated, and statistical analysis was performed. I) PCV2 copies in the inguinal lymph nodes and spleen. The respective tissues were collected from each pig and total DNA was isolated 14 d after PCV2 challenge. The number of PCV2 copies was determined using RT‐qPCR and compared to that of known standards. J) Body weight is plotted as a percentage of the average initial weight on day 0 and changes over 14 d are shown. (B‐F, H, I) n = five pigs. Data are expressed as the mean ± SEM. *P* values were calculated using one‐way ANOVA with Tukey's multiple comparison test. (G) n = five pigs. Representative images from each group are shown. (B‐F, H, I) Asterisks indicate significant differences between groups linked by horizontal lines. ***, *P* < 0.001; **, *P* < 0.01; *, *P* < 0.05. (J) ***, *P* < 0.001; **, *P* < 0.01, compared with strains rSC0139(pS‐Cap^ACA−^) and rSC0140(pS‐Cap^ACA−^). The experiments were performed twice. The results from both experiments were similar and the data were pooled for analysis.

Pigs were orally inoculated with the relevant strains and assessed for acquired immune cytokine levels, antibody titers, and protective immune responses (**Figure** **7**A). Porcine peripheral blood mononuclear cells (PBMCs) inoculated with PBNV‐expressing rSC0140(pS‐Cap^ACA−^) showed significantly higher expressions levels of IL‐4, IFN‐γ, and GrzB compared with those in pigs inoculated with non‐PBNV‐expressing rSC0139(pS‐Cap^ACA−^) (Figure 7B). These results indicated that the PBNV system enhanced the ability of the vector to induce antigen‐specific T‐cell responses to delivered foreign antigens. The titers of Cap‐specific IgG, IgG1, and IgG2 in the sera of pigs inoculated with rSC0140(pS‐Cap^ACA−^) were significantly higher than those in pigs inoculated with rSC0139(pS‐Cap^ACA−^) (Figure 7C,E). These findings indicated that PBNV enhanced the *Salmonella* vector‐induced specific humoral immune response to foreign antigens. Likewise, the Cap‐specific IgA titers in nasal washes from pigs inoculated with rSC0140(pS‐Cap^ACA−^) were significantly higher than those in pigs inoculated with rSC0139(pS‐Cap^ACA−^) (Figure 7D), indicating that the PBNV system enhances the *Salmonella* vector‐induced antigen‐specific mucosal immune response. In addition, the IgG2 titers induced by either rSC0140(pS‐Cap^ACA−^) or rSC0139(pS‐Cap^ACA−^)were significantly higher than the IgG1 titers induced by these strains (Figure 7E). This finding indicated that the CDA‐producing *Salmonella* vector induced a foreign antigen‐specific Th1‐biased immune response that was unaffected by the PBNV system. Serum neutralizing antibody titers against PCV2 in pigs inoculated with rSC0140(pS‐Cap^ACA−^) were significantly higher than those in pigs inoculated with rSC0139(pS‐Cap^ACA−^) (Figure 7F), indicating that PBNV enhanced the protective immune response. Following PCV2 challenge, pigs inoculated with rSC0140(pS‐Cap^ACA−^) showed significantly lower PCV2 signals (brown) in the inguinal lymph nodes (Figure 7G,H) and viral copy numbers (Figure 7I) compared with those in pigs inoculated with rSC0139(pS‐Cap^ACA−^). Additionally, pigs inoculated with rSC0140(pS‐Cap^ACA−^) exhibited significantly less weight loss than those inoculated with rSC0139(pS‐Cap^ACA−^) (Figure 7J). These results demonstrated that the PBNV system markedly improved the protective efficacy against PCV2 infection mediated by *Salmonella* vectors delivering Cap‐NPs. In consideration of the both adaptive and innate immune responses in the system, we further analyzed the immunotype induced by the PBNV strain. Specifically, we measured the levels of the innate immune marker IFN‐β and the titers of the adaptive immune marker Cap‐specific antibodies at days 1, 5, 9, 13, 17, and 21 post‐inoculation. The expression of IFN‐β reached a high level (>150 pg mL^−1^) by day 1 post‐inoculation. Over time, its levels gradually declined, dropping below 50 pg mL^−1^ after day 9 (Figure S6A, Supporting Information). Cap‐specific antibodies were undetectable within the first 5 days. However, between days 5 and 21 post‐inoculation, the Cap‐specific IgG titers steadily increased, surpassing 15 000 by day 21 (Figure S6B, Supporting Information). These data suggest that the antiviral response induced by the PBNV system is initially dominated by innate immunity (up to approximately day 9), followed by a shift toward adaptive immunity later in the period.

## 3 Discussion

Although the inherent lymphatic colonization ability of *Salmonella* vector systems is considered a significant advantage of this vaccine platform,^[^ ^31^, ^32^ ^]^ the limited expression of foreign antigens or cargo within *Salmonella* cells greatly restricts their in vivo delivery.^[^ ^32^ ^]^ This limitation becomes even more complex when the cargo is NPs because NP assembly typically requires specific buffering conditions.^[^ ^38^ ^]^ The bacterial periplasmic space is thought to be a “Garden of Eden” for NP self‐assembly, owing to its unique oxidative environment that facilitates proper protein folding.^[^ ^36^ ^]^ However, current strategies directly limit periplasmic targeting.^[^ ^12^, ^37^, ^39^ ^]^ In this study, we observed that the expression of foreign proteins in host cells inoculated with rSC0140, which expresses the PBNV system, was significantly enriched in the periplasmic space compared with that in cells inoculated with rSC0139, which does not express the PBNV system (Figure 1E,F; Figure S1B,C, Supporting Information). Furthermore, this efficient periplasmic localization translated into robust NP assembly (Figure 2A; Figure S2, Supporting Information) and effective in vivo delivery (Figure 2H,I). Because of the dual role of PBNV in enhancing target protein production through the SIRV system and facilitating cytoplasmic cargo release,^[^ ^33^ ^]^ we hypothesized that the robust periplasmic‐targeting effect of PBNV promotes the self‐assembly of NPs, making bacterial cells highly efficient for synthesizing NP‐vaccines. Previous studies have extensively reported chimeric technologies using Cap‐, L1‐, and CTBTti‐NPs for foreign antigen presentation.^[^ ^12^, ^34^, ^40^ ^]^ Therefore, our strategy has the potential to be used as an efficient platform for producing multivalent NP vaccines that target other pathogens.

Although the catalytic properties of DacA have been reported,^[^ ^21^ ^]^ the enzymatic reactions require suitable catalytic conditions and recombinant substrates.^[^ ^41^ ^]^ The availability of ATP in the cytoplasm of *Salmonella* as a substrate for DacA enzymatic reactions remained unknown. In this study, we observed that *Salmonella* vectors expressing DacA catalyzed the production of CDA (Figure 3A‐C), demonstrating that *Salmonella* vectors can serve as efficient biofactories for the biosynthesis of STING agonists. Intriguingly, this exogenous catalytic reaction was enhanced by increasing the concentration of DacA. When PBNV was used to elevate the expression levels of DacA (Figure 1C,D), the production of CDA was correspondingly increased (Figure 3A‐C). Therefore, our strategy represents a purely biological platform for STING agonist production, significantly reducing costs and shortening production cycles. ATP is essential for fundamental biological processes,^[^ ^42^ ^]^ and serves as a readily accessible raw material for biochemical reactions.^[^ ^42^, ^43^ ^]^ Hence, beyond *Salmonella*, this strategy for synthesizing STING agonists has versatile applications. For instance, this system could be potentially adapted and used in mammalian,^[^ ^44^ ^]^ insect,^[^ ^45^ ^]^ or yeast,^[^ ^46^ ^]^ cell expression systems among others.

The cytoplasmic localization of STING dictates that the corresponding agonists must be transported there for a reaction to occur.^[^ ^19^, ^24^ ^]^ Despite the identification of numerous cGAS‐STING pathway agonists, delivering them specifically to the host cell cytoplasm remains a challenge because of their poor membrane permeability.^[^ ^20^, ^47^ ^]^ In this study, we observed that *Salmonella* vectors could deliver loaded CDA specifically into the intracellular space (Figure 3A‐C). Moreover, PBNV significantly enhanced the cytoplasmic targeting and delivery of CDA (Figure 3A‐C). Therefore, PBNV not only optimized the delivery and release of protein antigens, but also improved the cytoplasmic‐targeted delivery and release of small‐molecule agonists. Using the example of small‐molecule STING agonists, we highlighted the advantages of the PBNV system‐based *Salmonella* vector for targeted delivery, particularly for those targeting cytosolic surveillance pathways.

STING agonists are small nucleotide‐based molecules with high polarity, which are prone to rapid degradation and clearance via enzymatic processes after intravenous injection.^[^ ^47^, ^48^ ^]^ We observed the persistence of CDA in the lymphatic system of mice for up to 7 d (Figure 4B), indicating that the PBNV‐based CDA delivery strategy mediates efficient and durable in vivo delivery. Indeed, the synthesis of foreign cargo within *Salmonella* is continuous and is accompanied by the persistent colonization of bacteria in vivo.^[^ ^32^ ^]^ Additionally, *Salmonella* utilizes a unique mechanism to extend its survival inside cells involving the formation of *Salmonella*‐containing vacuoles (SCVs),^[^ ^49^ ^]^ which confer *Salmonella* cells with the ability to evade lysosomal degradation.^[^ ^49^, ^50^ ^]^ Therefore, our strategy of utilizing PBNV for delivering STING agonists mitigated their premature clearance, ensuring effective in vivo delivery.

Recurring pandemics of infectious diseases are attributed to the continual mutation and recombination of their genomes, leading to the emergence of novel pathogens.^[^ ^51^, ^52^, ^53^ ^]^ This characteristic renders high‐cost pharmaceuticals and vaccines obsolete before they can be realized.^[^ ^14^, ^53^ ^]^ In this study, compared with the non‐PBNV‐expressing rSC0139, cells treated with the PBNV‐expressing rSC0140 showed significantly elevated activation levels of the STING‐ISG pathway (Figure 3D‐I; Figure S4A‐F, Supporting Information), which translated into efficient inhibition of infection by H1N1, H5N1, H7N9, and PCV2 viruses (Figures S3B‐J and S4G‐I, Supporting Information). Correspondingly, in both mice and pigs, the PBNV‐expressing strains demonstrated stronger antiviral responses against H1N1, H5N1, H7N9, and PCV2 than the non‐PBNV‐expressing strains (Figures 5B‐I and 7G‐J). These results strongly demonstrated that the PBNV‐targeted delivery of CDA activates the host immune system, resulting in broad‐spectrum antiviral effects against both DNA and RNA viruses. This is not limited to the viruses examined in this study. The STING‐ISG pathway has been shown to be involved in the host defense against various viruses,^[^ ^54^ ^]^ including SARS‐CoV‐2,^[^ ^55^ ^]^ herpesviruses, influenza viruses,^[^ ^56^ ^]^ and HIV,^[^ ^57^ ^]^ among other pandemic pathogens. Therefore, the PBNV system can be applied in multiple antiviral infection models, particularly against emerging and unknown viral pathogens. Our findings highlighted the strategies for targeting innate immune pathways in vaccine design, as antiinfection effects based on innate immune responses are typically broad and rapid.^[^ ^58^ ^]^

Most current investigations on STING agonists are confined to murine models^[^ ^20^ ^]^ with limited studies being conducted on large animals such as pigs. Notably, the phenotype of a novel STING agonist observed in murine models may not necessarily translate to other species. For example, the STING agonist DMXAA was effective in mice but not in humans owing to structural differences in STING between species.^[^ ^59^ ^]^ In this study, we observed that the delivery of CDA via *Salmonella* vectors significantly activated STING‐dependent innate immune responses in both murine and porcine macrophages (Figure 3D‐I; Figure S4A‐F, Supporting Information). This immune response translated into the maturation of APCs, significant T‐cell activation, elevated humoral immune responses, and higher protective antibody titers in both mice and pigs (Figures 6B‐G and 7B‐F). These results demonstrated that our delivery strategy enabled CDA to exert strong adaptive immunostimulatory effects in both mouse and pig models, indicating its broad species applicability. Although CDA has been shown to possess immunomodulatory functions,^[^ ^21^, ^23^ ^]^ few studies have explored its reactivity as a vaccine adjuvant. Our findings highlighted the superior adaptive immunostimulatory capabilities of CDA delivered via *Salmonella* vectors in vivo, paving the way for the development of related vaccine adjuvants. Therefore, we concluded that the PBNV system combats infection not only by enhancing the innate immune response but also by improving the adaptive immune response.

In summary, we presented a strategy that utilizes *Salmonella* vectors to achieve the production and self‐assembly of NPs, autocatalytic synthesis of small‐molecule STING agonists, and their targeted in vivo delivery. Importantly, the operation of the entire platform was purely biological, representing a vivid and comprehensive production process. Owing to its high level of engineering, our system can be directionally modified in the future to support diverse in vivo cargo delivery systems and vaccine development.

## 4 Experimental Section

### Animals and Ethics Statement

All experimental procedures were performed in accordance with the guidelines for laboratory animal welfare and ethics by the Jiangsu Administrative Committee. License numbers SYXK (SU) 2021‐0026 and SCXK (SU) 2017‐0007 were issued by the Jiangsu Province's Department of Science and Technology for animal monitoring. Six‐week‐old female BALB/c mice were purchased from the Comparative Medicine Centre of Yangzhou University (Yangzhou, China). Three‐week‐old female mixed‐breed Landrace white piglets were purchased from Jiangsu Lihua Animal Husbandry Co. Ltd. (Changzhou, China). Neither mice nor piglets tested positive for *Salmonella* outer membrane protein (OMPs) or Cap.

Bacterial strains, plasmids, and culture conditions The bacterial strains and plasmids used in this study are listed in Table S1 (Supporting Information). Plasmids pS‐Cap^ACA−^, pS‐L1^ACA−^, and pS‐CTBTri^ACA−^ were derived from pS0018 and carried a PCV2‐*cap* (GenBank ID: 2943185), HPV16‐*l1* (GenBank ID: 1489082), and an engineered *ctbtri* gene,^[^ ^12^ ^]^ respectively, without the ACA sequence. All oligonucleotides and gene fragments were commercially synthesized. Primers used are listed in Table S2 (Supporting Information). Suicide vectors were used to introduce mutations into *Salmonella* via conjugation, as previously described.^[^ ^33^ ^]^ Using the gene sequence of DacA from *Listeria monocytogenes* strain NH1 (GenBank ID: 1 358 004 970) as a framework, a *dacA* nucleotide sequence was designed that did not contain the ACA sequence (*dacA* ^ACA−^, 822 bp), while keeping the amino acid sequence of DacA unchanged. The *dacA* ^ACA−^ sequence was placed under the control of the P_lac_ promoter to form the P_lac_ *dacA* ^ACA−^ expression cassette. Primers used to construct the Δ*recF*:P_lac_ *dacA* ^ACA−^ suicide vector are listed in Table S1 (Supporting Information). Suicide vectors were used for the creation of unmarked deletion and deletion‐insertion mutations in *Salmonella* strains through conjugational transfer by employing the donor strain χ7213, as previously reported.^[^ ^33^ ^]^ *E. coli* and *Salmonella* strains were grown in Luria‐Bertani (LB; Oxoid) broth, Nutrient Broth (NB; BD Difco), or plated onto LB agar at 37 °C. When necessary, chloramphenicol (25 mg mL^−1^), kanamycin (50 mg mL^−1^), ampicillin (100 mg mL^−1^), 2,6‐diaminopimelic acid (50 mg mL^−1^), arabinose (0.5% w/v), and D‐lactose (1% w/v) were used.

### Generation of Polyclonal Antibodies Against DacA

Recombinant His6‐DacA was synthesized in BL21(DE3) cells carrying the plasmid pET28a‐DacA. Purification of recombinant proteins was carried out using a Ni‐agarose chromatography medium kit (Solarbio) following the manufacturer's instructions. For generating polyclonal antibodies, rabbits were immunized with the appropriate recombinant proteins.

### Bacteria Subcellular Fractionation

To assess the subcellular localization of synthesized Cap, L1, or CTBTri in the respective strains, cultures in the mid‐exponential growth phase were collected by centrifugation at 4 °C. Periplasmic fractions were prepared using a modified lysozyme‐osmotic shock method as previously described.^[^ ^37^ ^]^ In brief, cultures were grown in LB medium until they reached the logarithmic phase and then induced with 0.5 mM isopropyl‐β‐D‐thiogalactopyranoside (IPTG, Sigma Aldrich) for 3 h. The supernatant was filtered and retained for analysis of secreted proteins. Equal volumes of periplasmic, cytoplasmic, and supernatant fractions as well as total protein lysate samples were separated on a 10% SDS‐PAGE gel and transferred to a nitrocellulose membrane for western blot analysis. The respective protein bands were densitometrically quantified using the ImageJ software (NIH) and normalized to the value of GroEL.

### Phenotypes of Foreign Proteins in PBNV

Relevant bacterial strains were cultured to the logarithmic phase, and then induced with the addition of 0.5 mM IPTG for 3 h. The induced samples were then subjected to western blot analysis using anti‐DacA, anti‐Cap, anti‐HPVL1 (Abcam), anti‐Cap, cholera toxin beta monoclonal (CTBTri‐specific, Thermo Fisher Scientific), or anti‐GroEL (Abcam) antibodies, as previously described.^[^ ^33^ ^]^ Imprinting was performed using ChemiDoc XRS+ (BioRad). Figures are representative of at least three independent experiments. The respective protein bands were densitometrically quantified using the ImageJ software (NIH) and normalized to the value of GroEL.

### Transmission Electron Microscopy (TEM)

Observation of bacterial periplasm: Bacterial samples were preserved in a 2.5% (v/v) glutaraldehyde solution for 12 h at 4 °C. Subsequently, the samples were rinsed thrice with PBS for 15 min each. The samples were then treated with a 2% (v/v) solution of osmic acid (Sigma Aldrich) for 2 h, followed by three washes with PBS for 10 min each. The specimens were dehydrated in a sequential series of ethanol solutions (50, 70, 80, 90, and 100% [v/v]) for 15 min each. Subsequently, they were immersed in a mixture of SPI‐Pon 812 resin (SPI) and acetone in ratios of 1:1, 1:2, or 1:3. Finally, the specimens were soaked overnight in 100% SPI‐Pon 812 resin. Following a polymerization process at 65 °C for 36 h, the samples were cut into sections and treated with uranyl acetate (Sigma Aldrich) and lead citrate (Sigma Aldrich) prior to examination under a TEM.

### Observation of NPs

*Salmonella* strains were cultivated in LB medium supplemented with 0.5% (w/v) L‐arabinose (Sigma Aldrich) and 0.1% (w/v) D‐mannose (Sigma Aldrich) at 37 °C. The culture was allowed to enter its logarithmic growth phase (OD_600_ of 0.6–0.8), and was then stimulated with 0.3 mM IPTG for 10 h at 30 °C. Cells were disrupted by sonication, and the resulting mixture was separated by centrifugation at 15 and 600 × *g* for 10 min. The cell lysate from the appropriate strains was applied to a copper grid (200 mesh) and left for 10 min at 37 °C. Afterward, the grids were immersed in a solution of 3% phosphotungstic acid (PTA) for 10 min, and any extra liquid was removed using dry cotton. Finally, the samples were observed under a Tecnai 12 TEM electron microscope (Philips).

### BMDCs Preparation

BMDCs were generated from the bone marrow of BALB/c mice using a well‐established method.^[^ ^60^ ^]^ In summary, BMDCs were grown in RPMI 1640 medium (HyClone) supplemented with 10% (v/v) FBS, 100 U mL^−1^ penicillin‐streptomycin, 500 U mL^−1^ IL‐4 (Sigma Aldrich), and 1000 U mL^−1^ GM‐CSF (Sigma Aldrich). Cultural media were refreshed bidaily. Cells underwent differentiation for 5–8 d on 15‐cm Petri plates. The resulting cell population consisted of a minimum of 85% CD11c^+^ cells and was placed in tissue culture‐treated plates 1 d prior to infection.

### Antigen Release from Bacteria

BMDCs were inoculated with the appropriate strains in 6‐well plates at a multiplicity of infection (MOI) of 10. Following gentamycin administration and subsequent washing to eliminate extracellular bacteria, BMDCs were cultivated for 24 h. At 12 h after infection, a group of dishes was fixed, permeabilized, and stained using anti‐Cap and anti‐CD11c‐FITC (Abcam) antibodies. Rabbit anti‐mouse IgG‐PE (Abcam) was used as a secondary antibody. The samples were examined on a FACSAria SORP cytometer (BD Biosciences).

### Colonization of Mice with PBNV

Prior to oral inoculation with *Salmonella* strains, female BALB/c mice, aged six weeks, were subjected to a 4‐h fasting from both food and water. The strains were then transferred to fresh culture media (LB) containing 0.5% (w/v) arabinose and 0.2% (w/v) mannose. The cultures were grown at 37 °C for 16 h until reaching an OD_600_ of 0.85. The relevant strains were orally administered to mice (1 ± 0.2 × 10^9^ CFU in 20 µL of a PBS suspension). Mice were provided with food and water for 45 min. Peyer's patches, spleen, and liver were collected at specific time intervals using aseptic techniques. To evaluate the process of colonization and long‐term persistence, tissues were homogenized and plated on LB agar containing 0.5% (w/v) arabinose.

### Analysis of In Vivo Delivery of NPs

For mice: strains rSC0139 with either pS‐Cap^ACA−^ or pS0018 and rSC0140 with either pS‐Cap^ACA−^ or pS0018 were orally administered to mice (1 ± 0.2 × 10^9^ CFU suspended in 20 µL PBS). At 7 d after inoculation, the spleens of mice were isolated and subjected to an indirect immunofluorescence assay (IFA) to detect Cap‐NP‐specific signals. For pigs: the corresponding strains were orally administered to pigs at a dose of 5 ± 0.2 × 10^9^ CFU per pig. At 7 d after inoculation, the inguinal lymph nodes of pigs were isolated and subjected to IFA to detect Cap‐NP‐specific signals. Tissues were fixed in 10% formalin, embedded in paraffin, and 3‐µm sections were cut. Tissue sections were subjected to antigen retrieval in citrate buffer and blocked with 5% bovine serum albumin (BSA, Sigma Aldrich). The sections were incubated overnight with primary antibody against Cap, and staining was visualized using a goat anti‐mouse IgG‐FITC coupling antibody (Abcam). DAPI (Sigma Aldrich) was used to stain the cell nuclei.

### LC‐MS Analysis of CDA

LC‐MS analysis was performed on an Acquity UPLC I‐class/VION IMS QTOF mass spectrometer (Waters), using an EC 150/2.0 NUCLEODUR C18 pyramid chromatography column (3 µm). The flow rate was set to 0.4 mL min^−1^, the column temperature to 30 °C, and the injection volume to 5 µL. The mobile phase consisted of 0.2% (v/v) aqueous formic acid and methanol. Each sample was analyzed within 5 min. The mass spectrometry parameters were as follows: ion spray voltage at 5500 V, ion source temperature at 350 °C, declustering voltage at 90 V, intake voltage at 7 V, ion source gas 1 at 40 psi, ion source gas 2 at 40 psi, and curtain gas at 20 psi. The number of quantitative ion pair was 659/330 (parent ion/daughter ion).

### Analysis of the Cytoplasmic Targeting of CDA

RAW264.7 cells were infected with rSC0138, rSC0139(pS0018), rSC0140(pS0018), rSC0139(pS‐Cap^ACA−^), and rSC0140(pS‐Cap^ACA−^) according to the specified protocol.^[^ ^23^ ^]^ At 12 h after infection, the medium was removed, and cells were permeabilized with digitonin (5 µg mL^−1^, Sigma Aldrich) in a buffer solution containing 30 mM HEPES (Sigma Aldrich), 50 mM KCl (Solarbio), 2 mM MgCl2 (Solarbio), 0.1 mM dithiothreitol (Solarbio), 60 mM sucrose (Sigma Aldrich), and 0.5% BSA (Sigma Aldrich). Cell samples were diluted with 50% (v/v) acetonitrile and centrifuged at 10 000 RPM for 20 min. The supernatant was filtered through a 0.22 µm ultrafiltration filter (Millipore), and then vacuum dried. The reconstituted CDA was ultrasonically processed for 30 min at 25 °C in 0.5 mL of 50% (v/v) acetonitrile. After centrifugation at 10 000 RPM for 20 min, the supernatant was collected for LC‐MS analysis.

### Analysis of In Vivo CDA Delivery

Mice were randomly divided into six groups (20 mice/group) and inoculated with 1 ± 0.2 × 10^9^CFU (rSC0138, rSC0139(pS0018), rSC0140(pS0018), rSC0139(pS‐Cap^ACA−^), or rSC0140(pS‐Cap^ACA−^). Peyer's patches, spleens, and livers were aseptically collected at indicated time points. Tissues were weighed and homogenized in a final volume of 1 mL methanol (CDA is highly soluble in methanol). The homogenized samples were centrifuged at 10 000 RPM for 10 min. The supernatant was collected and CDA levels were measured using LC‐MS.

### CRISPR‐Cas9 Knockout

CRISPR‐Cas9 technology was used for generating genetic knockouts. Specific double‐stranded oligonucleotides, designed to target a particular sequence, were inserted into the lenti‐CRISPR‐V2 vector. This vector was cotransfected together with packaging plasmids into HEK293 cells. Viruses were obtained 2 d after transfection and used to infect RAW264.7 or 3D4/21 cells. Cells were subjected to puromycin screening (1 µg mL^−1^, Sigma Aldrich) for 7 d, starting 1 d after transfection. Afterward, cells were diluted twice in 96‐well plates to create oligoclonal cell lines for gene deletion. Phenotypic responses to relevant stimuli were evaluated to further establish the functional impact of the protein knockdown, as described below. Serial dilutions were used to generate individual clonal knockout cells and confirmed using Sanger sequencing. Table S2 (Supporting Information) contains the gRNA sequences targeting mouse cGAS, mouse TL4, and pig TLR4.

### Macrophage Analysis

RAW264.7, cells were infected with *Salmonella* strains following a previously described protocol.^[^ ^60^ ^]^ In summary, bacterial cultures were washed three times with PBS and resuspended in DMEM supplemented with 10% FBS. The suspension was added to cell monolayers, centrifuged at 1000 RPM for 10 min to synchronize host cell adhesion, and then incubated at 37 °C with 5% CO_2_ for 1 h. Extracellular bacteria were eliminated by incubation with gentamycin for 1 h, after which cells were washed twice with PBS and maintained in DMEM. Supernatants were collected 12 h after infection to measure IFN‐β levels. Protein concentrations were determined using a Micro BCA Protein Kit (Solarbio) and equal amounts of protein were subjected to western blot analysis. Samples were detected using anti‐STING (Abcam), anti‐phospho‐STING (Cell Signaling Technology), anti‐TBK1 (Abcam), anti‐phospho‐TBK1 (Cell Signaling Technology), anti‐IRF3 (Cell Signaling Technology), anti‐phospho‐IRF3 (Cell Signaling Technology), ISG15 (Thermo Fisher Scientific), or viperin (Abcam) antibodies. Values were normalized to that of β‐actin detected using an anti‐β‐actin antibody (Abcam). The procedure for detecting members of the STING‐ISG pathway in 3D4/21 cells was identical to that in RAW264.7 cells. Phospho‐STING (Ser365) antibodies were used for RAW264.7 cells, whereas Phospho‐STING (Ser366) antibodies were employed for 3D4/21 cells (Table S3, Supporting Information). cGAMP was transfected into cells as a positive control using the X‐tremeGENE HP DNA transfection reagent (Roche) at a concentration of 100 ng mL^−1^.

### Analysis of Antiviral Efficiency In Vitro

For IAVs, wild‐type, cGAS^−/−^, or TLR4^−/−^ RAW264.7 cells were stimulated with the corresponding strains for 12 h before the transfer of media to MDCK cells. MDCK cells were infected with IAVs (MOI 0.01) for 24 h. The media were used to determine the IAV load. Briefly, 50 000 MDCK cells were seeded in each well of a 96‐well plate. The following day, the sample was added to 12 replicates and serially diluted. The results were read at 48 h using light microscopy, and the 50% tissue culture infective dose (TCID_50_) was calculated using the Reed‐Muench method.^[^ ^61^ ^]^ For PCV2, wild type, cGAS^−/−^, and TLR4^−/−^ 3D4/21 cells were stimulated with the corresponding strains for 12 h before the transfer of media to PK15 cells. PK15 cells were infected with PCV2 (MOI 0.1) for 24 h. The medium was used to determine the PCV2 load. The TCID_50_ of PCV2 was determined using IFA, as described in a previous study.^[^ ^62^ ^]^ The virus was serially diluted 10‐fold. PK15 cells, grown in 96‐well plates (Corning), were exposed to 100 µL of PCV2 per well for 2 h. After incubation, the unbound virus was removed by washing the cells thrice with PBS. Cells were then fixed with a methanol‐acetone solution for 45 min at 4 °C, followed by three PBS washes. Anti‐Cap monoclonal antibody, diluted 1:200 in PBS, was added to the plates and incubated for 2 h at 37 °C. After washing thrice with PBS, cells were incubated with goat anti‐mouse IgG‐FITC coupling antibody (Abcam) for 1 h at 37 °C in the dark, followed by another three PBS washes. Cells were stained with DAPI (Sigma Aldrich) to visualize the nuclei and observed under an inverted fluorescence microscope (Olympus).

### Analysis of STING‐ISG Pathway In Vivo

Mice were randomly divided into six groups (5 mice/group) and inoculated with 1 ± 0.2 × 10^9^CFU of rSC0138, rSC0139(pS0018), rSC0140(pS0018), rSC0139(pS‐Cap^ACA−^), rSC0140(pS‐Cap^ACA−^) or 20 µL PBS. The production of IFN‐β in mice seracollected 24 h after inoculation was analyzed. Peyer's patches were collected from mice at 7 d after inoculation. Tissues were weighed, homogenized in a final volume of 1 mL PBS, and incubated with tissue cell lysis buffer (Solarbio) on ice for 15 min. The samples were subjected to western blot analysis using anti‐STING (Abcam), anti‐phospho‐STING (Cell Signaling Technology), anti‐TBK1 (Abcam), anti‐phospho‐TBK1 (Cell Signaling Technology), anti‐IRF3 (Cell Signaling Technology), anti‐phospho‐IRF3 (Cell Signaling Technology), anti‐ISG15 (Thermo Fisher Scientific), or anti‐viperin (Abcam) antibodies.^[^ ^33^ ^]^ For all western blot analyses, β‐actin was used as a loading control. Pigs were randomly divided into six groups (5 pigs per group) and inoculated with 5 ± 0.2 × 10^9^ CFU of the relevant strains. The production of IFN‐β in mice or pigs sera collected 24 h after inoculation was analyzed. Apart from the target organ being the inguinal lymph nodes, and the use of the corresponding detection antibodies (Table S3, Supporting Information), the procedure for detecting members of the STING‐ISG pathway in pigs was identical to that in mice.

### Analysis of Antiviral Efficacy In Vivo

Mice were randomly assigned to six groups (40 mice per group) and inoculated with either 1 ± 0.2 × 10^9^ CFU of the relevant strains or 20 µL PBS. At 7 d after inoculation, immunized and control mice were challenged with an intranasal instillation of 10 × LD_50_ of IAVs, except for the H7N9 virus, which was administered at 100 × LD_50_.^[^ ^63^, ^64^, ^65^ ^]^ Body weight and survival rates were monitored daily for 14 d after the challenge. Mice that lost more than 20% of their body weight were euthanized. When necessary, mouse lungs were fixed in 10% (v/v) formalin buffer. Lung tissues were embedded in paraffin wax, sectioned at 3‐µm thickness, and stained with hematoxylin and eosin (H&E) using a staining kit (Solarbio) according to the manufacturer's instructions. Pathological alterations were assessed under an optical microscope. Pathological scores for each mouse histological section were assigned as follows: 0, normal; 1, congested; 2, interstitial thickening, 3 inflammatory cell infiltration in the bronchial submucosa; and 4, extensive inflammatory cell infiltration.

### Immunohistochemistry of Lungs

Lung tissues were fixed in 10% formalin, embedded in paraffin, and 3‐µm sections were cut. Tissue sections were subjected to antigen retrieval in citrate buffer and blocked with 5% BSA (Sigma Aldrich). The sections were incubated overnight with primary antibodies against ISG15 (Thermo Fisher Scientific) and viperin (Abcam). Staining was visualized using the appropriate secondary antibodies (Abcam), while DAPI (Sigma Aldrich) was used to stain the cell nuclei. The corresponding antibodies are listed in Table S3 (Supporting Information).

### BMDC Analysis

BMDCs were incubated with the relevant strains at a MOI of 10 for 48 h. Supernatants were collected to measure IL‐6 and IL‐12p70 levels. Cells were stained with CD11c‐FITC, CD86‐PE, and CD40‐APC (Abcam) and analyzed using a FACSAria SORP cytometer (BD Biosciences).

Cytokine quantification Cytokine levels in cell culture supernatants or mouse samples were measured using commercial ELISA kits. Kits for porcine IFN‐β, porcine IL‐1β, porcine TNF‐α, mouse IFN‐β, mouse IL‐1β, mouse TNF‐α were from Abcam, while those for mouse IL‐6 and IL‐12p70 were from BD Biosciences. All procedures were performed according to the manufacturer's instructions.

### Confocal Fluorescence Microscopy

Minor alterations were made to previously reported procedures while preparing samples for fluorescence microscopy.^[^ ^33^ ^]^ In brief, the relevant bacteria were cultured to the logarithmic growth phase and induced with 0.5 mM IPTG for 3 h. After centrifugation at 10 000 RPM for 3 min, the bacteria were washed thrice with PBS. Following fixation with 4% paraformaldehyde (Solarbio), bacteria were permeabilized with a solution containing digitonin (2 µg mL^−1^, Sigma Aldrich), 20 mM KCl (Solarbio), and 2 mM MgCl2 (Solarbio), allowing the specific binding of antibodies to antigens within the bacteria. An anti‐Cap monoclonal antibody was added to the bacterial suspension and incubated at 4 °C for 12 h, followed by washing thrice with PBS. The stained specimens were affixed to a glass slide and imaged using an SP8 Laser Scanning Confocal Microscope (Leica) at a 1000× magnification. The LAS X software (Leica) was used to deconvolve the medial focus planes.

### Immunofluorescence Assay of CD8^+^ T‐Cells in the Spleen

Mice were inoculated with the relevant strains. Spleens were harvested from immunized mice at 7 d after the booster immunization. The spleens were fixed in 10% formalin (Solarbio), embedded in paraffin (Leica), and sectioned at 3‐µm thickness. The sections were deparaffinized, rehydrated, and treated with 3% hydrogen peroxide (Solarbio) for 30 min to block endogenous peroxidase activity. Cell dissociation was performed by incubating sections in 1% trypsin (Sigma Aldrich) at 37 °C for 15 min. The sections were subjected to antigen retrieval in citrate buffer and blocked with 5% BSA (Sigma Aldrich). The sections were then incubated overnight with PE‐conjugated anti‐mouse CD8 alpha (Abcam) at 4 °C., and DAPI (Sigma Aldrich) was used to stain the cell nuclei.

### ELISPOT Assays

The ELISPOT plates were coated with anti‐mouse IFN‐γ (Abcam), IL‐4 (Abcam), or GrzB (Abcam) antibodies according to the manufacturer's instructions. The plates were stimulated with 10% (v/v) ethanol (Solarbio) and cultured for 30 min in the presence of 10% (v/v) FBS (HyClone). To identify IL‐4‐secreting CD4^+^ T‐cells, IFN‐γ‐secreting or GrzB‐secreting CD8^+^ T‐cells from immunized mice, CD4^+^ or CD8^+^ T‐cells were isolated from the spleen using the CD4^+^ or CD8^+^ T‐cell Isolation Kit (Miltenyi) according to the manufacturer's instructions. T‐cells were placed in a 96‐well plate at a density of 1 ± 0.2 × 10^6^ cells per well and incubated at 37 °C with 5% CO_2_ for 48 h in the presence of Cap protein. Cells were extracted and cultured with biotinylated antibodies that specifically target mouse IFN‐γ (BD Biosciences), IL‐4 (Abcam), or GrzB (Thermo Fisher Scientific). Prior to injecting the streptavidin‐HRP conjugate (Solarbio), the plates were rinsed thrice with PBS. The plates were developed using a ready‐to‐use AEC substrate (Solarbio). After drying, the number of generated spots was measured using an immune spot reader (Cellular Technology Ltd.,). Data were collected from three separate wells.

### Pig Administrations and Challenges

Pigs were randomly divided into five groups (5 pigs/group) and inoculated through the oral route with 1 ± 0.2 × 10^10^ CFU of rSC0139(pS0018), rSC0140(pS0018), rSC0139(pS‐SaoA^ACA−^), rSC0140(pS‐SaoA^ACA−^) or 5 mL PBS, followed by a booster immunization at three weeks. Sera and nasal wash samples were collected at three and five weeks following first inoculation and stored at ‐20 °C. The levels of nasal wash IgA, serum IgG, IgG1, and IgG2 against Cap were assayed using ELISA, as described above.^[^ ^62^ ^]^ Pigs were challenged with an intranasal instillation of 1000 × TCID_50_ PCV2, five weeks after the first inoculation. Body weight and survival were monitored daily for 14 d after challenge. Pigs were observed twice daily for clinical symptoms and mortality 14 d after challenge. When necessary, inguinal lymph nodes were fixed in 10% (v/v) formalin buffer (Solarbio) for subsequent immunohistochemistry (IHC) analysis and stored at ‐80 °C for subsequent nucleic acid extraction. The data from two independent replicates of the protection tests were similar and aggregated for analysis.

### IHC Analysis

IHC was conducted on 3‐µm‐thick tissue sections to detect PCV2 antigen. The sections were deparaffinized, rehydrated, and treated with 3% hydrogen peroxide (Solarbio) for 30 min to block endogenous peroxidase activity. Cell dissociation was performed by incubating with 1% trypsin (Sigma Aldrich) at 37 °C for 15 min. After washing, sections were incubated with BSA‐PBS for 1 h at 37 °C to block nonspecific binding. The sections were then incubated with Cap monoclonal antibodies for 2 h at 37 °C. After incubation with primary antibodies, the sections were treated with anti‐mouse horseradish peroxidase (HRP)‐conjugated antibodies and stained using a DAB Stain Kit (Solarbio) according to the manufacturer's guidelines.

Enzyme‐linked immunosorbent assay (ELISA) Polystyrene 96‐well flat‐bottom microtiter plates (Corning) were coated with 50 ng per well of pure Cap. In each well, 100 µL of serially diluted sample was added in triplicate and then incubated for 2 h at 37 °C. The plates were then treated with rabbit anti‐pig IgG‐HRP (Thermo Fisher Scientific), mouse anti‐pig IgG1‐HRP (Thermo Fisher Scientific), mouse anti‐pig IgG2‐HRP (Bio‐Rad), or goat‐anti pig IgA‐HRP coupling antibody (Thermo Fisher Scientific). The plates were prepared using a TMB single‐component substrate kit (Solarbio) and the reaction was terminated using a 5% H_2_SO_4_ solution. The absorbance was measured at 450 nm using an automated ELISA plate reader (Model EL311SX; Biotek). Absorbance levels that were 2.1 times higher than the baseline values of naïve serum were considered positive.

### Acquired Immune Cytokines in Pigs

Seven days after booster immunization, porcine splenocytes were obtained from the immunized piglets. Cells were cultured in RPMI‐1640 (HyClone) supplemented with 10% FBS (HyClone) and IL‐2 (Abcam, 100 U/mL). Cells were then placed in wells at a density of 1 × 10^6^ cells per well, along with Cap protein, and incubated in a humidified incubator at 37 °C and 5% CO_2_ for 72 h. Subsequently, cells were collected and total RNA was extracted. The extracted RNA was used to assess the levels of IL‐4, IFN‐γ, and GrzB transcripts using the primers listed in Table S2 (Supporting Information). The relative expression of target genes was calculated by normalizing to the expression of pig β‐actin using the 2^^‐ΔΔCq^ method.

### Quantitative Real‐Time PCR (qRT‐PCR)

The Triquick Reagent^®^ RNA extraction kit (Solarbio) was used to extract total RNA from cells according to the manufacturer's instructions. Subsequently, 1 µg of total RNA was converted into complementary DNA (cDNA) through reverse transcription to be used in quantitative real‐time PCR (qRT‐PCR). qRT‐PCR reactions were conducted on a 7500 Fast Real‐Time PCR Instrument (ABI) using the KiCqStart^®^ SYBR^®^ Green qPCR ReadyMix (Sigma Aldrich).

PCV2 neutralization assay The neutralization assay was conducted according to a previously described procedure.^[^ ^62^ ^]^ Prior to the neutralization experiment, sera samples were subjected to heat inactivation at 56 °C for 30 min. Afterward, 50 µL of inactivated serum was diluted by a factor of two in DMEM (Hyclone) and combined with an equivalent amount of PCV2 (100 × TCID50; TCID50 = 10^−6.15^). The mixture was then incubated at 37 °C for 30 min. PK‐15 cells cultured in 96‐well plates were exposed to a mixture of virus and serum. Cells were then incubated at 37 °C for 48 h. Afterward, cells were fixed with 0.3% (v/v) Triton X‐100 (Solarbio) and 4% (w/v) paraformaldehyde (Solarbio) for 10 min. Subsequently, cells were incubated at 37 °C for 1 h with 1% (w/v) BSA (Sigma Aldrich) to inhibit any nonspecific binding. After washing thrice with PBS, cells were consecutively incubated with pig serum and FITC‐conjugated goat anti‐mouse IgG (Abcam) for 2 h. Cells were examined under an inverted fluorescence microscope (Olympus). The neutralizing antibody titer was determined by calculating the reciprocal of the greatest dilution that provided full protection to PK‐15 cells.

### Quantification of PCV2 in Tissue Samples

DNA was extracted from mouse tissue samples using a GenElute™ mammalian genomic DNA kit (Sigma Aldrich) following the manufacturer's instructions. DNA content was determined using a NanoDrop 3300 spectrophotometer (Thermo Fisher Scientific). The DNA was kept at ‐80 °C. The number of PCV2 *rep* gene copies was measured by quantitative real‐time PCR (qRT‐PCR) using the KiCqStart^®^ SYBR^®^ Green qPCR ReadyMix (Sigma Aldrich) according to the manufacturer's protocol. Three replicates were conducted for each group. An absolute quantitative approach was used to analyze the data. Serial dilutions ranging from 0 to 10^9^ copies of the PCV2 plasmid were prepared using a 7500 Rapid Real‐Time PCR apparatus (Applied Biosystems) to obtain standard values. The mean Ct values were used to determine the number of *rep* gene copies, which were then divided by the weight of the tissue and converted into viral copies per mg of tissue.

### Statistical Analyses

Comparisons across groups were conducted using the Mann‐Whitney U test with the GraphPad Software, Inc (Dotmatics). All data, except for the animal survival rate, are presented as the mean ± standard deviation. Survival after challenge was assessed using the log‐rank (Mantel‐Cox) test. A value of *P* < 0.05 was considered statistically significant.

## Conflict of Interest

The authors declare no conflict of interest.

## Author Contributions

Y.‐a.L. was responsible for implementing the assays, interpreting the data, and writing the first draft. Y.F., W.L., and Y.S. were involved in certain assays. Y.Z., S.W., R.C.III, and H.S. were involved in revising and editing. Y.‐a.L. and H.S. were involved in experiment design, were responsible for the interpretation of the data, and for monitoring the exploration process. All authors read and approved the final manuscript.

## Supporting information



Supporting Information

## Data Availability

The data that support the findings of this study are available from the corresponding author upon reasonable request.
