# Supplementary material for: A Purely Biomanufactured System for Delivering Nanoparticles and STING Agonists
Source: Adv Sci (Weinh). 2024 Nov 26;12(3):2408539. doi: 10.1002/advs.202408539 (PMC11744655; doi:10.1002/advs.202408539)
Supplement: Supplementary file 1 — Supporting Information [file ADVS-12-2408539-s001.pdf]

ADVANCED SCIENCE
Open Access

Supporting Information

for *Adv. Sci.*, DOI 10.1002/advs.202408539

A Purely Biomanufactured System for Delivering Nanoparticles and STING Agonists

Yu-an Li, Yi Feng, Wenjing Li, Yuqin Zhang, Yanni Sun, Shifeng Wang, Roy Curtiss III and Huoying Shi**

A purely biomanufactured system for nanoparticles and STING agonists delivering

Yu-an Li[1, 2], Yi Feng[1, 2], Wenjing Li[1, 2], Yuqin Zhang[1, 2], Yanni Sun[1, 2], Shifeng Wang[3], Roy Curtiss III[3*], Huoying Shi[1, 2, 4 *]

[1] *College of Veterinary Medicine, Yangzhou University, Yangzhou 225009, Jiangsu, China*

[2] *Jiangsu Co-innovation Center for the Prevention and Control of Important Animal Infectious Diseases and Zoonoses, Yangzhou, China*

[3] *Department of Infectious Diseases and Immunology, College of Veterinary Medicine, University of Florida, Gainesville, FL 32611-0880, USA*

[4] *Joint International Research Laboratory of Agriculture and Agri-Product Safety, Yangzhou University (JIRLAAPS), Yangzhou, China*

Figure S1

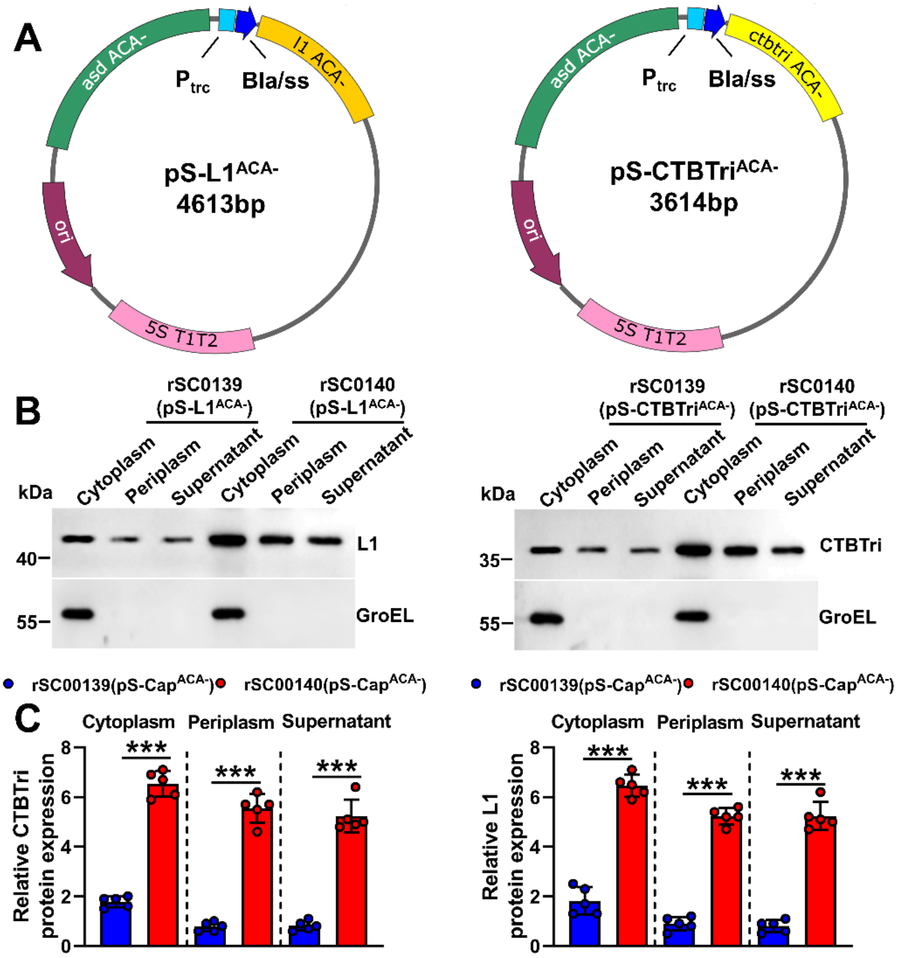

Figure S1. The PBNV system improves periplasmic targeting of foreign proteins. (A) Plasmid maps of recombinant plasmid pS-L1^{ACA-} (left) and recombinant plasmid pS-CTBTri^{ACA-} (right). Subcellular location analyses of L1 or CTBTri expressed in *Salmonella*. Representative images (B) and densitometric measurements (C) are shown. n = five biological replicates per group, one representative picture is shown for each group. Data are expressed as the mean ± SEM. *P* values were calculated by one-way ANOVA with Tukey's multiple comparison test. Asterisks indicate significant differences between groups linked by horizontal lines. ***, $P < 0.001$. The experiments were performed twice. The results from both experiments were similar, and the data were pooled for analysis.

Figure S2

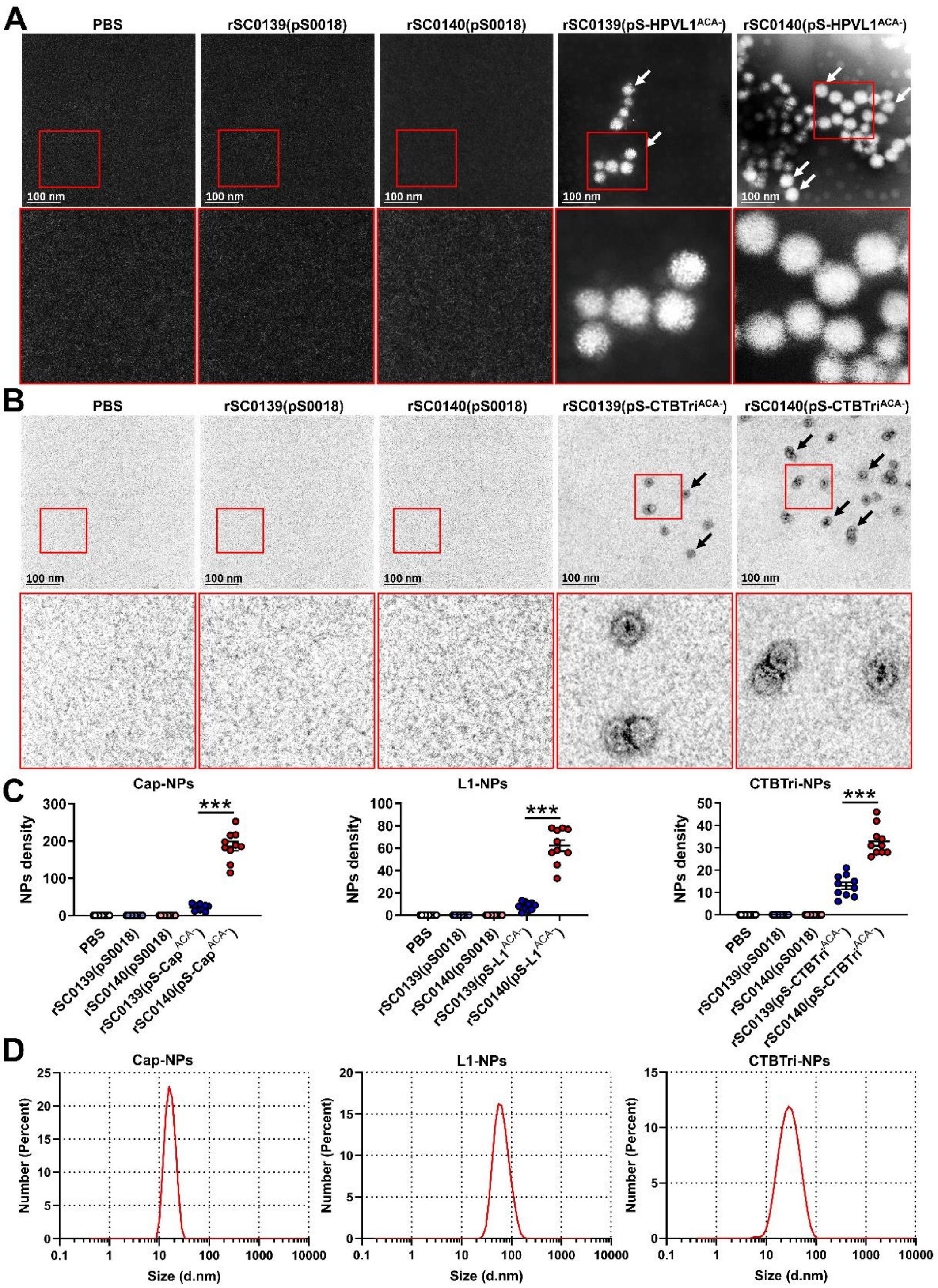

Figure S2. The PBNV system enhances NPs' yield.

(A) TEM analyses of PCV2-VLPs from lysates of PBS, rSC0139(pS0018), rSC0140(pS0018), rSC0139(pS-Cap^{ACA-}), or rSC0140(pS-Cap^{ACA-}) (bars = 100 nm). Arrows indicates L1-NPs. (B) TEM analyses of PCV2-VLPs from lysates of PBS,

rSC0139(pS0018), rSC0140(pS0018), rSC0139(pS-Cap^{ACA-}), or rSC0140(pS-Cap^{ACA-}) (bars = 100 nm). Arrows indicates CTBTri-NPs. (C) Ten fields were randomly selected to count the number of particles in the field. (D) DLS analysis of the NPs. The Cap-NPs, L1-NPs, and CTBTri-NPs were characterized by DLS. The result showed the diameter of Cap-NPs Cap-NPs, L1-NPs, and CTBTri-NPs were about 17 nm, 25 nm, and 53 nm, respectively. (A, B, D) n = three biological replicates per group, one representative picture is shown for each group. Data are expressed as the mean ± SEM. *P* values were calculated by one-way ANOVA with Tukey's multiple comparison test. Asterisks indicate significant differences between groups linked by horizontal lines. ***, $P < 0.001$. The experiments were performed twice. The results from both experiments were similar, and the data were pooled for analysis.

Figure S3

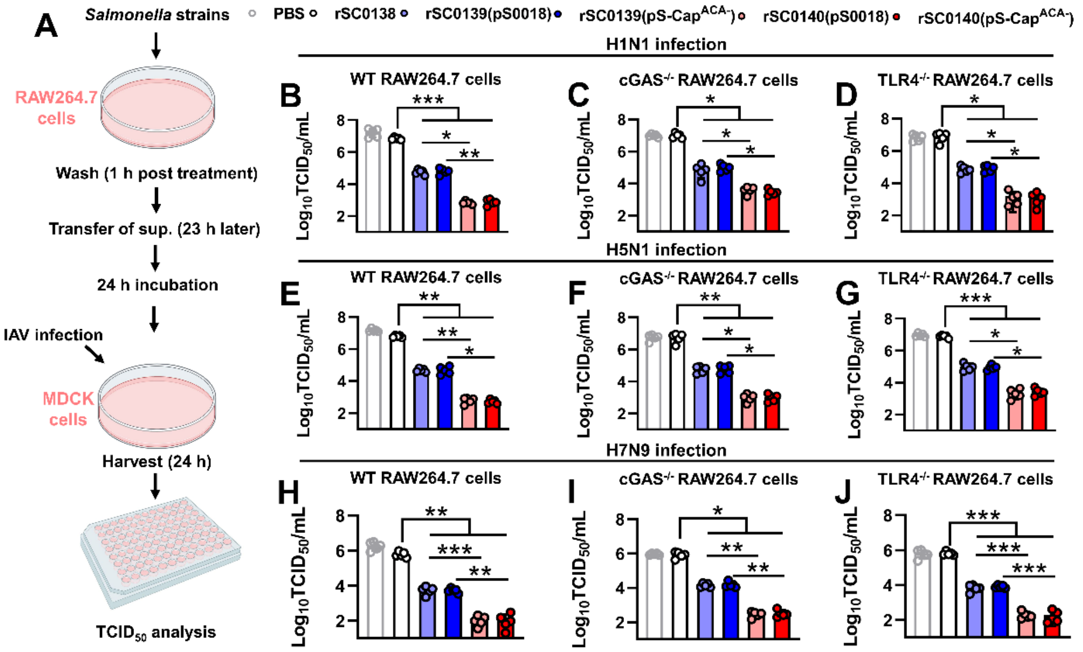

Figure S3. The PBNV inducing a broad-spectrum antiviral response.

(A) RAW267.7 cells were stimulated with *Salmonella* strains for 24 hours before the media were transferred to MDCK cells. MDCK cells were infected 24 hours later with IAVs (MOI 0.1) for 24 hours and the media were used to determine the IAVs load. The H1N1 virus grown in triplicate in WT RAW264.7 cells (B), cGAS KO RAW264.7 cells (C), and TLR4 KO RAW264.7 cells (D) and pre-infected for 12 h

with rSC0139 and rSC0140 carrying either pS0018 or pS-Cap^{ACA-}, respectively. The H5N1 virus grown in triplicate in WT RAW264.7 cells (E), cGAS KO RAW264.7 cells (F), and TLR4 KO RAW264.7 cells (G) and pre-infected for 12 h with rSC0139 and rSC0140 carrying either pS0018 or pS-Cap^{ACA-}, respectively. The H7N9 virus grown in triplicate in WT RAW264.7 cells (H), cGAS KO RAW264.7 cells (I), and TLR4 KO RAW264.7 cells (J) and pre-infected for 12 h with rSC0139 and rSC0140 carrying either pS0018 or pS-Cap^{ACA-}, respectively. n = five biological replicates per group. Data are expressed as the mean ± SEM. *P* values were calculated by one-way ANOVA with Tukey's multiple comparison test. Asterisks indicate significant differences between groups linked by horizontal lines. ***, *P* < 0.001; **, *P* < 0.01; *, *P* < 0.05. The experiments were performed twice. The results from both experiments were similar, and the data were pooled for analysis.

Figure S4

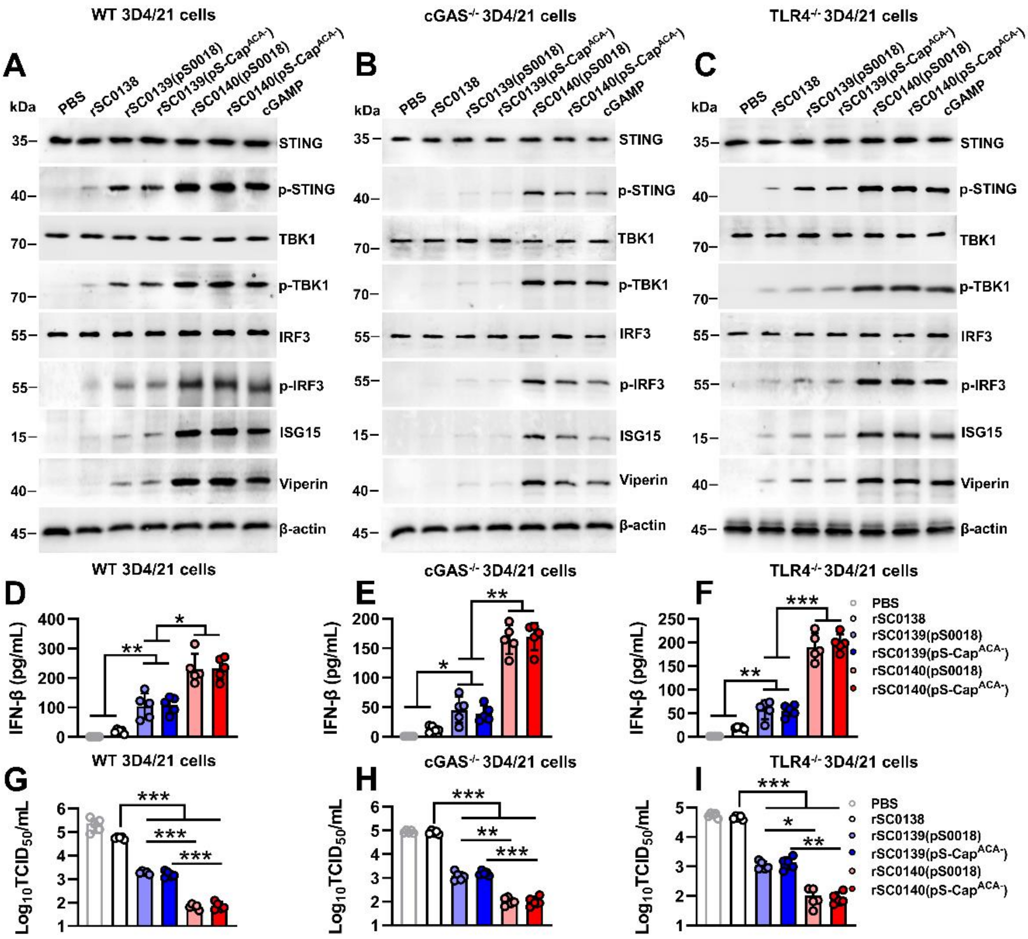

Figure S4. The PBNV inducing activation of the STING-ISGs axis and an antiviral response in pig cells.

STING, p-STING, TBK1, p-TBK1, IRF3, p-IRF3, ISG15, and Viperin expression in WT 3D4/21 cells (A), cGAS KO 3D4/21cells (B), and TLR4 KO 3D4/21 cells (C) were assessed using western blotting at 12 h post-infection with indicated strains. For all western blot analyses, β-actin was used as a loading control. Secretory IFN-β levels in were assessed 12 h post-infection in WT 3D4/21 cells (D), cGAS KO 3D4/21 cells (E), and TLR4 KO 3D4/21 cells (F) using ELISA. The PCV2 grown in triplicate in WT 3D4/21 cells (G), cGAS KO 3D4/21 cells (H), and TLR4 KO 3D4/21 cells (I) and pre-infected for 12 h with rSC0139 and rSC0140 carrying either pS0018 or pS-Cap^{ACA-}, respectively. n = five biological replicates per group. Data are expressed as the mean ± SEM. *P* values were calculated by one-way ANOVA with Tukey's multiple comparison test. Asterisks indicate significant differences between groups linked by horizontal lines. ***, $P < 0.001$; **, $P < 0.01$; *, $P < 0.05$. The experiments were performed twice. The results from both experiments were similar, and the data were pooled for analysis.

Figure S5

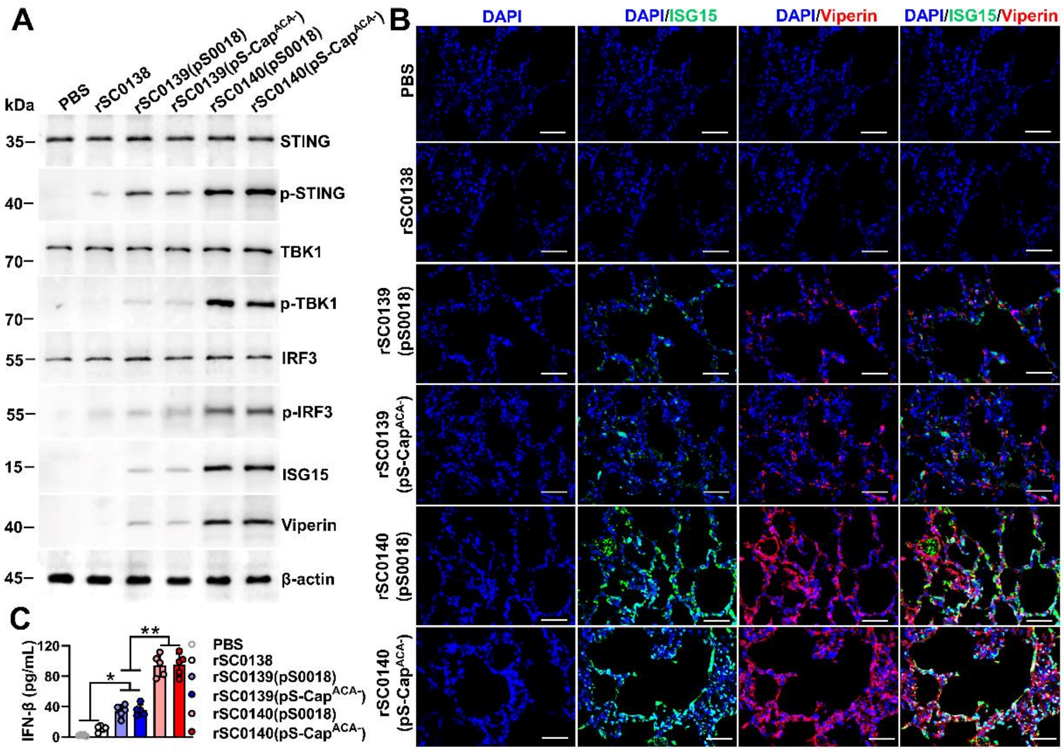

Figure S5. The PBNV activates the STING-ISGs pathway, inducing a systemic antiviral response in pigs.

(A) STING, p-STING, TBK1, p-TBK1, IRF3, p-IRF3, ISG15, and Viperin expression in inguinal lymph nodes of pigs were assessed using western blotting at 12 h post-infection with indicated strains. For all western blot analyses, β-actin was used as a loading control. (B) Immunofluorescence analysis. Pigs were inoculated with the relevant strains. Lungs were isolated from pigs at 24 h after inoculation, respectively. Paraffin sections of the lungs were stained for Viperin (red) and ISG15 (green). DAPI (blue) marks the nuclei (bars = 50 nm). (C) Secretory IFN-β levels in pig serum were assessed by ELISA 24 h post-primary immunization. (A) n = five pigs. One representative picture is shown for each group. (B, C) n = five pigs. Data are expressed as the mean ± SEM. *P* values were calculated by one-way ANOVA with Tukey's multiple comparison test. Asterisks indicate significant differences between groups linked by horizontal lines. **, $P < 0.01$; *, $P < 0.05$. The experiments were performed twice. The results from both experiments were similar, and the data were pooled for analysis.

Figure S6

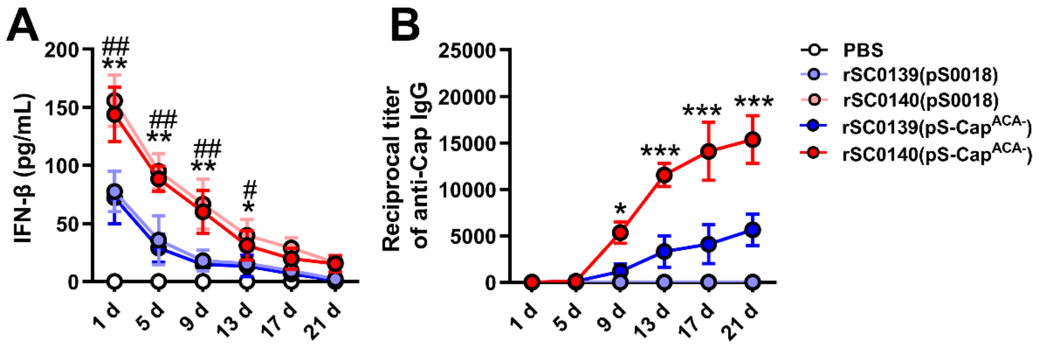

Figure S6. Immunotype analysis induced by the PBNV strain.

(A) Levels of IFN-β in mouse serum after inoculation with the PBNV strain. (B) Titers of Cap-specific IgG antibodies in mouse serum after inoculation with the PBNV strain. Mice were orally inoculated with rSC0140(pS-Cap^{ACA-}), and serum

samples were collected at the indicated time points. ELISA was used to analyze the levels of IFN-β and Cap-specific IgG antibodies in the serum, assessing the innate and adaptive immune responses. ###, $P < 0.001$; ##, $P < 0.01$; #, $P < 0.05$, compared with strains rSC0139(pS0018) and rSC0140(pS0018); ***, $P < 0.001$; **, $P < 0.01$; *, $P < 0.05$, compared with strains rSC0139(pS-Cap^{ACA-}) and rSC0140(pS-Cap^{ACA-}). n = 5 mice; The experiments were performed twice. The results from both experiments were similar, and the data were pooled for analysis.

Figure S7

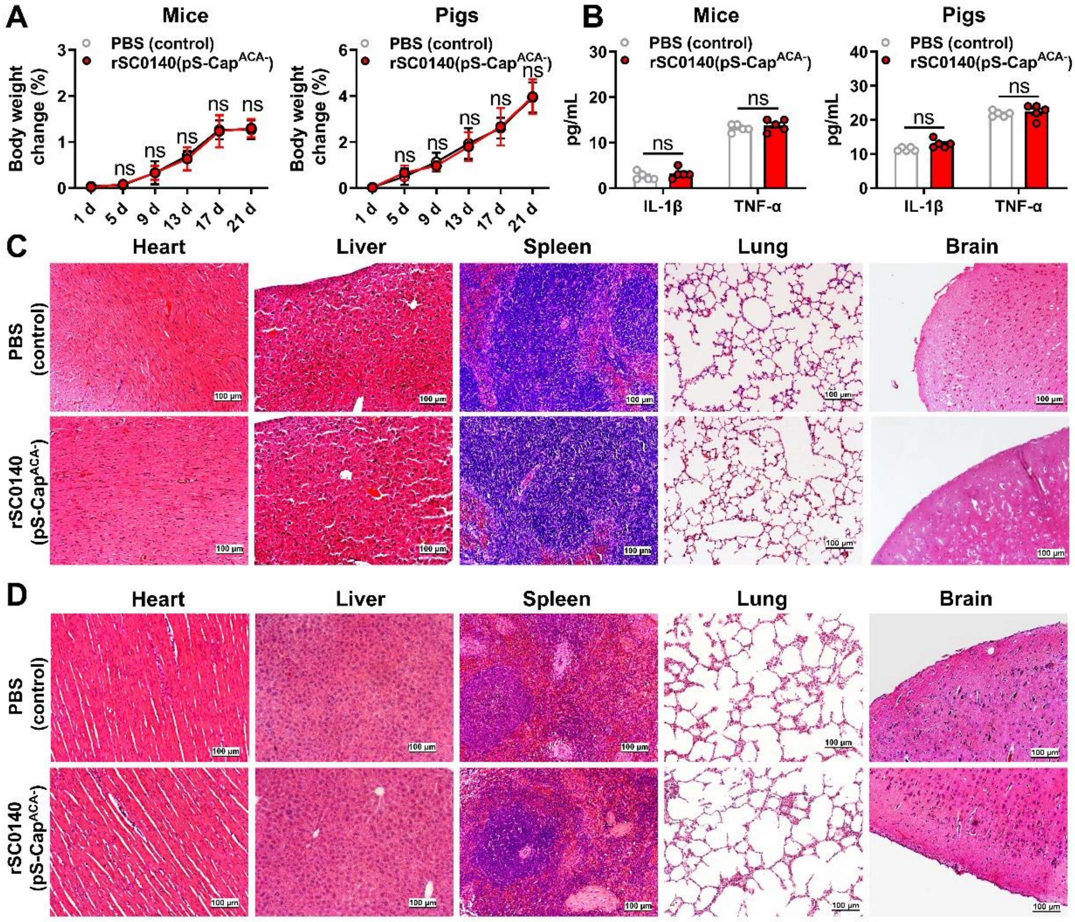

Figure S7. Safety estimation of PBNV strain.

(A) Weight changes in mice and pigs inoculated with PBNV strain. No abnormal body weight was found in the treated group compared with that of the control group. (B) Cytokine profiles (IL-1β, TNF-α) in serum of mice and pigs at 2 days after inoculation. No significant difference was found between the treated group and control group, indicating that the PBNV strain has no systemic toxicity. (C) HE

staining of pathological sections of mice organs at 7 days after inoculation. No organic damage or acute inflammation was observed in the histological sections of the heart, liver, spleen, lung, and brain, which further confirmed the safety of the vaccine. n = five mice and the control was mice treated with PBS. (D) HE staining of pathological sections of pigs organs at 7 days after inoculation. No organic damage or acute inflammation was observed in the histological sections of the heart, liver, spleen, lung, and brain, which further confirmed the safety of the PBNV strain. n = three pigs and the control was pigs treated with PBS. ns, no significant difference. The experiments were performed twice. The results from both experiments were similar, and the data were pooled for analysis.

Table S1 Strains, plasmids, and vectors

Bacterial Strains	Source	Reference
C78-3 Wild type *Salmonella* Choleraesuis, virulent, CVCC79103	Lab. stock	N/A
rSC0115, C78-3 + Δ*relA*::*araC* P_{araBAD} *lacI* TT, Δ*endA*::*araC* P_{araBAD} *mazE* TT	Lab. stock	[33]
rSC0117, C78-3 + Δ*relA*::*araC* P_{araBAD} *lacI* TT, Δ*endA*::*araC* P_{araBAD} *mazE* TT, Δ*cysG*:P_{lac} *mazF*	Lab. stock	[33]
rSC0138, C78-3 + Δ*relA*::*araC* P_{araBAD} *lacI* TT, Δ*manA*, Δ*endA*::*araC* P_{araBAD} *mazE* TT, Δ*cysG*:P_{lac} *mazF*, Δ*recF*	This study	N/A
rSC0139, C78-3 + Δ*relA*::*araC* P_{araBAD} *lacI* TT, Δ*manA*, Δ*endA*::*araC* P_{araBAD}	This study	N/A

mazE TT, Δ*recF*:P_{lac} *dacA*$^{ACA-}$, Δ*asdA*		
rSC0140, C78-3 + Δ*relA*::*araC* P_{araBAD} *lacI* TT, Δ*manA*, Δ*endA*::*araC* P_{araBAD} *mazE* TT, Δ*cysG*:P_{lac} *mazF*, Δ*recF*:P_{lac} *dacA*$^{ACA-}$, Δ*asdA*	This study	N/A
χ7213	Lab. stock	[61]
BL21	Invitrogen	Cat# EC0114
DH5α	Invitrogen	Cat# 18258012
PCV2, Wild type, virulent	Lab. stock	[62]
H1N1, PR8 strain, virulent	Lab. stock	[63]
H5N1, CK10 strain, virulent	Lab. stock	[64]
H7N9, JTC11 strain, virulent	Lab. stock	[65]
Plasmids	Source	Reference
pS0018	Lab. stock	[33]
pS-Cap^{ACA-}	This study	N/A
pS-L1^{ACA-}	This study	N/A
pS-CTBTri^{ACA-}	This study	N/A
pRE112 *sacB mobRP4 R6K oriV oriT Cm*^{r}	Lab. stock	N/A
pYA3736: pRE112 Δ*asdA33*	Lab. stock	N/A
pRE112 Δ*recF*:P_{lac} *dacA*$^{ACA-}$	This study	N/A

LentiCRISPRv2	AddGene	Cat# 52961
LentiCRISPRv2-mouse-cGAS	This study	N/A
LentiCRISPRv2-mouse-TLR4	This study	N/A
LentiCRISPRv2-pig-TLR4	This study	N/A
psPAX2	AddGene	Cat# 12260

Talble S2 Primer or gRNA sequences

Primer name	Sequence (5'-3')
Construction of pRE112 Δ*recF*:P_{lac} *dacA*$^{ACA-}$	
dacA^{ACA-}-*Sal* I	GTCGACTCACTCGGACTTACCTCCT
dacA^{ACA-}-*Sph* I	GCATGCATGGACTTTTCCAATATGAGT
Kpn I-Δ*gyrB*-F	GGTACCCAGTGACGGCCAGGGGT
Sac I-*dnaN-R*	GAGCTCAGAATCCGGATAAACATCT
Construction of plasmid pS-Cap^{ACA-}	
Cap-*Smal* I	CGCCCGGGATGACGTATCCAAGGAGGCG
Cap-*Pst* I	GCCTGCAGTTAAGGGTTAAGTGGGGGGT
Construction of plasmid pS-L1^{ACA-}	
L1-*EcoR* I	ACGAATTCATGGTTGAGGTAGGTCGTG
L1-*Sal* I	GAGTCGACTCATACATATTCGCTCCCA
Construction of plasmid pS-CTBTri^{ACA-}	
CTBTri-*EcoR* I	TCGAATTCTGCAAAGGTGCTGACGGTG
CTBTri-*Sal* I	CAGTCGACGTAGTGCGGCGGCGGCGGT

Construction of plasmid pET28a-DacA	
DacA-*EcoR* I	TTGAATTCATGGACTTTTCCAATATGAGT
DacA-*Sal* I	GCAGTCGACTCACTCGGACTTACCTCCTT
Primer for q-RT PCR	
Pig β-actin	Forward: CGTCCACTCCGCCAGCAC
	Reverse: CTTGCTCTGGGCCTCGTC
Pig IL-4	Forward: GTCTCACCTCCCAACTGATC
	Reverse: ATGCACAGAACAGGTCATGT
Pig IFN-γ	Forward: TAGAAATTTTGAAGAATTG
	Reverse: CACTTTGATGAGTTCACTGA
Pig Grz-B	Forward: GAACTAAAAGAGAGCAAG
	Reverse: GATCAGGAACCCACCGCAC
PCV2-Rep	Forward: CTTCCGAAGACGAGCGCAAG
	Reverse: ATCAGTTCCTTTCGCTTTCT
gRNA sequence for CRISPR-Cas9 knockout	
Target to mouse cGAS	#1: GGAAGGGGCGTGGCCCCCTG
	#2: GCCGGACCGGGGCCCATACG
Target to mouse TLR4	#1: GGTTATTGTGGTAGTGTCTA
	#2: GTAAATGTCTTGTCAATCAG
Target to pig TLR4	#1: ACTACCAAGTTTACAGAAGC
	#2: TAGATCTGAGCTTCAATGAA

Table S3 Key resources table

Reagent or resource	Source or reference	Identifier
Antibodies		
Anti-HPV16 L1 antibody	Abcam	Cat# ab234305
Cholera toxin beta monoclonal antibody	Thermo Fisher Scientific	Cat# MA1-83519
Anti-STING antibody	Abcam	Cat# ab227705
Phospho-STING (Ser366) antibody, pig	Cell Signaling Technology	Cat# 50907
Phospho-STING (Ser365) antibody, mouse	Cell Signaling Technology	Cat# 72971
Anti-TBK1 antibody	Abcam	Cat# ab40676
Phospho-TBK1 (Ser172) antibody	Cell Signaling Technology	Cat# 5483
Anti-IRF3 antibody	Cell Signaling Technology	Cat# 11904
Phospho-IRF3 (Ser396) antibody	Cell Signaling Technology	Cat# 4947
Anti-ISG15 antibody, for mouse	Thermo Fisher Scientific	Cat# PA5-17461
Anti-ISG15 antibody, for pig	Thermo Fisher	Cat# MA5-15029

	Scientific	
Anti-Viperin antibody, for mouse and pig	Abcam	Cat# ab107359
Anti-mouse-IgG-PE coupling antibody	Abcam	Cat# ab7003
Anti-rabbit-IgG-FITC coupling antibody	Abcam	Cat# ab6717
Goat anti-mouse IgG-FITC coupling antibody	Abcam	Cat# ab6785
Anti-GroEL antibody	Abcam	Cat# ab90522
Anti-β-actin antibody	Abcam	Cat# ab8245
Rabbit anti-pig IgG-HRP	Thermo Fisher Scientific	Cat# PA1-28602
Goat anti-pig IgG1-HRP	Thermo Fisher Scientific	Cat# PA1-74421
Mouse anti-pig IgG2-HRP	Bio-Rad	Cat# MCA635
Goat anti-pig IgA-HRP coupling antibody	Thermo Fisher Scientific	Cat# PA1-84625
Rabbit anti-mouse IgG-PE detector	Abcam	Cat# ab7000
Biotinylated anti-mouse IFN-γ specific antibody	BD Biosciences	Cat# 554410
Biotinylated anti-mouse IL-4 specific antibody	Abcam	Cat# ab84269

Biotinylated anti-mouse GrzB specific antibody	Thermo Fisher Scientific	Cat# 13-8822-82
Anti-mouse IFN-γ antibody	Abcam	Cat# ab253676
Anti-mouse IL-4 antibody	Abcam	Cat# ab241928
Anti-mouse GrzB antibody	Abcam	Cat# ab317458
CD11c-FITC	Abcam	Cat# ab210308
CD40-APC	Abcam	Cat# ab272271
CD86-PE	Abcam	Cat# ab77226
CD8a-PE	Abcam	Cat# ab25498
$CD4^+$T cells isolation kit	Miltenyi	Cat# 130-104-453
$CD8^+$T cells isolation kit	Miltenyi	Cat# 130-104-075
Chemicals, Peptides, and Recombinant Proteins		
RPMI Medium 1640	Hyclone	Cat# SH30809.01
DMEM	Hyclone	Cat# SH30243.01
Luria-Bertani medium	Oxoid	Cat# CM0996B
Nutrient Broth	BD Difco	Cat# 231000
TMB single-component substrate kit	Solarbio	Cat# PR1200
Triquick Reagent® RNA extract kit	Solarbio	Cat# R1100

Fast SYBR Green Master Mix	Sigma	Cat# KCQS02
DAPI	Sigma	Cat# MBD0015
BSA	Sigma	Cat# B2064
FBS	Hyclone	Cat# SH30406.05
His-Cap protein	Lab. stock	[62]
Recombinant mouse GM-CSF	Sigma	Cat# SRP3201
Mouse IL-4	Sigma	Cat# SRP3211
Pig IL-2	Abcam	Cat# ab238302
Ni-agarose chromatography medium kit	Solarbio	Cat# P2010
Anti-Cap-monoclonal antibody	Lab. stock	N/A
Hematoxylin-Eosin (HE) staining kit	Solarbio	Cat# G1120
Mouse IFN-β ELISA kit	Abcam	Cat# ab252363
Porcine IFN-β ELISA kit	Abcam	Cat# ab273222
Mouse IL-6 ELISA set	BD Biosciences	Cat# 555240
Mouse IL-12p70 ELISA set	BD Biosciences	Cat# 555256
Experimental models: cell lines		
Mouse: RAW264.7	ATCC	Cat# TIB-71
HEK293	ATCC	Cat# CRL-11268
Pig: 3D4/21	ATCC	Cat# CRL-2843
MDCK	ATCC	Cat# CRL-2936

PK15	ATCC	Cat# CCL-33
Experimental models: organisms/strains		
Mouse: BALB/c	Beijing Vital River Laboratory Animal Co. Ltd.	Stock No: 211
Pig: landrace/white mixed breed pigs	Jiangsu Lihua Animal Husbandry Co., LTD	N/A
Software and algorithms		
GraphPad prism	Figure drawing and data analysis	https://www.graphpad.com/scientific-software/prism
Image-J	Band density analysis	NIH https://imagej.nih.gov/ij/
BioRender	Illustration Software	https://www.biorender.com/
